# HHLA2 activates c-Met and identifies patients for targeted therapy in hepatocellular carcinoma

**DOI:** 10.1186/s13046-025-03407-6

**Published:** 2025-05-20

**Authors:** Xubo Huang, Runya Fang, Yuqian Pang, Zhe Zhang, Jieru Huang, Yingchang Li, Tao Yuan, Yuyi Zeng, Ziying Yao, Silvia Vega-Rubín-de-Celis, Josephine Thinwa, Qisheng Zhang, Hao Shen, Jiahong Wang, Feng Shen, Yongjie Wei

**Affiliations:** 1 Guangzhou Institute of Cancer Research, The Affiliated Cancer Hospital, Guangzhou Medical University, Guangzhou, China; 2 School of Life Science, Guangzhou Medical University, Guangzhou, China; 3 Shenzhen Bay Laboratory & National-Regional Key Technology Engineering Laboratory for Medical Ultrasound, School of Biomedical Engineering, Shenzhen University Medical School, Shenzhen University, Shenzhen, China; 4 Clinical Research Institute, Department of Hepatic Surgery, Eastern Hepatobiliary Surgery Hospital & National Center for Liver Cancer, Shanghai, China; 5 Department of Hepatobiliary and Pancreatic Surgery, Tenth People’s Hospital of Tongji University, Shanghai, China; 6 Institute of Cell Biology (Tumor Research), University Hospital Essen, Virchowstr. Essen, Germany; 7 Department of Internal Medicine, University of Texas Southwestern Medical Center, Dallas, TX USA; 8 Shanghai Sino Organoid Lifesciences Co, Ltd, Shanghai, China

**Keywords:** Glycosylation, Angiogenesis, Invasion, NK cells, Immunosuppression, VEGFA

## Abstract

**Background:**

Hepatocellular carcinoma (HCC) is a highly aggressive malignancy with limited treatment options in advanced stages. While c-Met is a promising therapeutic target in HCC, identifying patients who will benefit from c-Met inhibitors remains a significant challenge. This study aimed to investigate the role of HHLA2, a B7 family member, in HCC and its potential as a liquid biopsy marker for c-Met inhibitor therapy.

**Methods:**

HHLA2 expression was analyzed in clinical HCC samples and public databases. In vitro studies using HCC cell lines assessed HHLA2’s impact on proliferation, migration, invasion, and angiogenesis. In vivo studies using mouse models (orthotopic xenografts and hydrodynamic tail vein injection) evaluated HHLA2’s role in tumor growth and metastasis. Mass spectrometry, co-immunoprecipitation, split-luciferase, and ELISA assays were used to investigate HHLA2-c-Met interactions. Patient-derived organoids (PDOs) were used to assess drug response. Statistical analyses included Student’s t-tests, ANOVA, and Cox regression.

**Results:**

HHLA2 was found to be upregulated in HCC and associated with advanced disease, aggressive clinicopathological features, and poor prognosis. HHLA2 interacted with and constitutively activated c-Met, leading to increased expression of MMP9 and VEGFA, enhancing HCC cell proliferation, invasion, and angiogenesis. HHLA2 also suppressed hepatic natural killer cell infiltration in vivo. Inhibition of c-Met with PHA665752 effectively reversed HHLA2-mediated tumor-promoting effects in vitro and in vivo. HHLA2 expression in HCC tissues correlated with c-Met phosphorylation, and HHLA2 could be detected in the serum of patients with high tumor HHLA2 levels. PDOs with high HHLA2 expression exhibited increased sensitivity to c-Met inhibition.

**Conclusions:**

HHLA2 acts as an oncogene in HCC by activating c-Met, promoting tumor progression and metastasis. HHLA2 expression correlates with c-Met activation and predicts poor prognosis in HCC patients. Importantly, HHLA2 can serve as a stratification marker for c-Met inhibitor therapy, potentially enabling a personalized approach to improve therapeutic outcomes in this challenging disease.

**Supplementary Information:**

The online version contains supplementary material available at 10.1186/s13046-025-03407-6.

## Introduction

The incidence of HCC is rapidly increasing, with estimates surpassing one million cases globally by 2040 [1] Despite early screening efforts, the majority of patients present with advanced, unresectable disease [2] Although treatment options including single-agent targeted therapies (e.g., sorafenib and lenvatinib), checkpoint inhibitor and target therapy combinations (e.g., atezolizumab plus bevacizumab), and dual-checkpoint inhibitors (e.g., durvalumab and tremelimumab) are available for advanced HCC, they only provide durable survival benefits for a small subset of patients and are often accompanied by adverse effects [3–5]. Therefore, there is a pressing need for effective therapies precisely targeting the root causes of HCC progression [6].

Among the various therapeutic targets explored, c-Met, a receptor tyrosine kinase activated by hepatocyte growth factor (HGF) has shown particular promise [7] While HGF/c-Met signaling is essential for normal liver development and repair, [8–11] its dysregulation drives HCC initiation and progression [12–15]. Approximately 50% of HCC cases exhibit c-Met aberrations such as overexpression, gene amplification, and mutations [16–18]. The presence of these aberrations is linked to increased tumor aggressiveness and a less favorable prognosis, highlighting the clinical relevance of c-Met in HCC.

While c-Met holds promise as a therapeutic target, evidenced by early pre-clinical successes, [19] its clinical translation has been hindered by significant challenges. The disappointing outcomes of two pivotal phase III tivantinib (ARQ 197) trials, a highly anticipated c-Met inhibitor, exemplify this hurdle [19–21]. These setbacks largely stem from difficulties in patient stratification. Optimal c-Met targeting relies on identifying tumors with “c-Met addiction”, where cancer cells depend on c-Met activation for survival and growth [22]. However, pinpointing true c-Met addiction has proven difficult. In tivantinib trials, due to the lack of a convenient and reliable biomarker for detecting c-Met activity (where c-Met phosphorylation represents the most direct detection method), immunohistochemical staining of c-Met overexpression in tumor tissue sections was used as an alternative approach. This approach, however, faces substantial limitations. Firstly, c-Met expression doesn’t always equate to activation, particularly in the absence of sufficient HGF levels. Secondly, c-Met can be activated through both canonical HGF-mediated and non-canonical pathways, making overexpression an unreliable indicator of functional activity [21, 6]. Consequently, relying solely on overexpression may exclude patients with c-Met activation driven by alternative mechanisms Moreover, the necessity for tissue biopsies to confirm c-Met overexpression introduces delays in treatment, potentially excluding rapidly progressing cases and biasing the study population toward less aggressive disease [23]. Effective implementation of selective c-Met inhibitors in HCC treatment requires a deeper understanding of c-Met activation mechanisms and reliable, non-invasive biomarkers for predicting therapeutic response.

HHLA2 is a recent addition to the B7 family of immune checkpoint molecules, distinguished by its evolutionary origin as a HERV-H endogenous retroviral protein and its primate-specific expression [24]. Initially discovered on hematopoietic cells, HHLA2 has subsequently been shown to be aberrantly upregulated in various solid tumors, including HCC, highlighting its potential role in oncogenesis. Although the precise mechanisms underlying HHLA2’s tumorigenic effects remain to be fully elucidated, growing evidence implicates it in promoting tumor proliferation, invasion, and metastasis [25–27]. These malignant properties may be attributed, in part, to HHLA2’s ability to interact with and modulate growth factor signaling pathways [25, 26]. Given the established role of the HGF/c-Met axis in HCC progression and the emerging oncogenic functions of HHLA2, exploring the potential interplay between these two pathways is warranted. Elucidating the molecular mechanisms underlying this interaction could reveal novel therapeutic targets and strategies for HCC.

This study identifies HHLA2 as a key regulator of c-Met signaling in HCC, promoting tumor progression and angiogenesis. HHLA2 interacts with and activates c-Met, and its expression correlates with advanced disease and poor prognosis. Serum HHLA2 levels correlate with tumor expression, potentially facilitating liquid biopsy for patient stratification. Furthermore, HHLA2-positive HCC models exhibit increased sensitivity to c-Met inhibitors, suggesting HHLA2 as both a therapeutic target and a predictive biomarker.

## Materials and methods

### Additional details are available in supplemental methods

#### Sex as a biological variable

This study exclusively used male mice due to the established higher incidence and more rapid progression of HCC in male mice compared to females I many commonly used mouse models, mirroring general trends observed in human HCC epidemiological. While this approach facilitates a more efficient study of HCC development, it limits the generalizability of our findings to female mice.

#### Omics data analysis

Publicly available datasets were used to explore HHLA2 expression and its clinical relevance and to investigate the potential link between HHLA2 expression and cancer cell response to therapy. Details of the databases and analysis protocols are provided in the Supplemental Methods.

#### Clinical samples

Two independent cohorts of HCC tissues were analyzed. Cohort 1 consisted of 176 paired HCC tumor and adjacent non-tumor tissue samples obtained from the Affiliated Cancer Hospital and Institute of Guangzhou Medical University. This cohort was used to analyze HHLA2 expression and its correlation with clinicopathological features. Cohort 2 consisted of 71 HCC tissue samples from the Eastern Hepatobiliary Surgery Hospital in Shanghai, China, and was used to evaluate the correlation between HHLA2 expression and phosphorylated Met (p-Met) levels.

#### Cell culture

Cells were cultured at 37 °C in a humidified incubator with 5% CO₂ in their respective media: SK-Hep-1, SMMC7721, and BEL7402 in RPMI 1640 medium; Hep3B in Eagle’s Minimum Essential Medium (EMEM); and SK-Hep-1, Hep3B, Huh7, MHCC97H, and HepG2 in Dulbecco’s Modified Eagle Medium (DMEM). All media were supplemented with 10% fetal bovine serum (FBS), except for DMEM used for HUVEC culture, which was supplemented with 20% FBS. Human umbilical vein endothelial cells (HUVECs) were cultured in DMEM supplemented with 20% FBS.

#### Animal models and in vivo experiments

Five-week-old male BALB/c nude mice and seven-week-old male C57BL/6 mice were obtained from the Guangdong Medical Laboratory Animal Center and housed under specific pathogen-free (SPF) conditions.

1. *Mouse Models*: Intrahepatic Xenograft Model: HCC cells were inoculated into the left lateral lobe of the liver of nude mice. 2)Lung Metastasis Model: Lung metastasis was assessed by bioluminescence imaging after tail vein injection of HCC cells into nude mice. 3) Hydrodynamic Co-expression Model: Hydrodynamic tail vein injection was used to deliver various combinations of plasmids encoding HHLA2 (or control), human NRAS, AKT, c-Met (human), and the sleeping beauty transposase (pT3/pCMV-SB) into 8-week-old C57BL/6 mice. While CMV activity is suboptimal in steady-state hepatocytes, the high efficiency of HDTVi drives sufficient transient expression of the CMV-driven Sleeping Beauty (SB) transposase to mediate effective genomic integration of the oncogenes within the critical early timeframe. This approach, supported by prior studies^28^ and the robust tumor formation observed in our model, leverages HDTVi’s unique delivery mechanism.
2. *C-Met Inhibitor Treatment*: Mice were treated intraperitoneally with the c-Met inhibitor PHA665752 (20 mg/kg) or vehicle (physiological saline) five times per week.
3. *Monitoring and Humane Endpoints*: Mice were monitored for changes in abdominal girth and signs of morbidity or discomfort. Animals were sacrificed at predetermined time points or when tumors reached a maximum size of 2 cm, in accordance with animal welfare guidelines. Further details on sample size determination and allocation are needed in the supplemental methods.

#### Organoid drug response assay

Patient-derived organoids were treated with either c-Met inhibitor PHA665752 (15 µM) or DMSO (vehicle control). After 48 h, cell viability and apoptosis were assessed using the Calcein-AM/PI Live/Dead Cell Double Staining Kit (Servicebio). Organoids were imaged using a high-content imaging analysis system (AMOview-100, Amoolo Biotech), with Calcein detected at 490/515 nm and propidium iodide (PI) at 535/617 nm. Fluorescence ratios were normalized to vehicle-treated controls. Lactate dehydrogenase (LDH) release in the organoid culture supernatant was measured after 48 h using the CyQUANT™ LDH Cytotoxicity Assay Kit (Thermo Fisher).

#### Statistics

Statistical analyses were performed using GraphPad Prism version 10. Unpaired or paired Student’s t-tests were used for two-group comparisons. One-way or two-way analysis of variance (ANOVA) followed by appropriate post-hoc tests (e.g., Tukey’s multiple comparisons test) were used for multiple group comparisons. Data normality was assessed using the Shapiro-Wilk test or other appropriate tests. Data are presented as mean ± standard deviation (SD) or median with interquartile range (IQR), as appropriate. P-values < 0.05 were considered statistically significant.

## Results

### Elevated HHLA2 expression correlates with advanced HCC and predicts poor patient survival

Analysis of publicly available transcriptomic data from GEO datasets (GSE190174, *n* = 5 HCC vs. 5 Normal; GSE64485, *n* = 50 HCC vs. 5 Normal) revealed significantly elevated HHLA2 mRNA levels in HCC tissues compared to normal tissues (Supplemental Fig. 1A). Utilizing data from The Cancer Genome Atlas Liver Hepatocellular Carcinoma project (TCGA-LIHC), we found this elevated HHLA2 mRNA expression (*n* = 371 samples with grade information) strongly correlated with advanced tumor grade (Supplemental Fig. 1B) and was associated with reduced overall survival (OS) (*n* = 371 samples with survival information, Supplemental Fig. 1C). Furthermore, multivariate Cox regression analysis of this TCGA cohort (based on *n* = 357 samples with complete covariate data) confirmed HHLA2 mRNA expression as an independent prognostic factor for OS (*p* = 0.022) (Supplemental Fig. 1D).

Further validation through immunohistochemical analysis of tumor tissues from our independent cohort of 176 HCC patients in Guangdong, China, confirmed significantly higher HHLA2 protein levels in tumors compared to adjacent non-tumor tissues (Supplemental Fig. 1E). Within this cohort (*n* = 176), high HHLA2 protein expression was significantly associated with aggressive clinicopathological features, including larger tumors (*p* < 0.001), increased vascular invasion (*p* = 0.016), advanced Barcelona Clinic Liver Cancer (BCLC) stages (*p* < 0.001), and higher recurrence and metastasis rates (*p* < 0.001) (Supplemental Fig. 1G, Supplemental Table 1). Both univariate and multivariate Cox regression analyses of this 176-patient cohort reinforced HHLA2 protein expression as an independent risk factor for poor prognosis (Hazard Ratio [HR] = 2.758; 95% Confidence Interval [CI] = 1.719–4.426; an HR > 1 is considered as poor prognosis) (Supplemental Fig. 1F, Supplemental Tables 1, 2). No significant associations were observed with other clinical parameters like gender, age, alpha-fetoprotein (AFP) levels, or hepatitis B status (Supplemental Table 1).

**Fig. 1:**
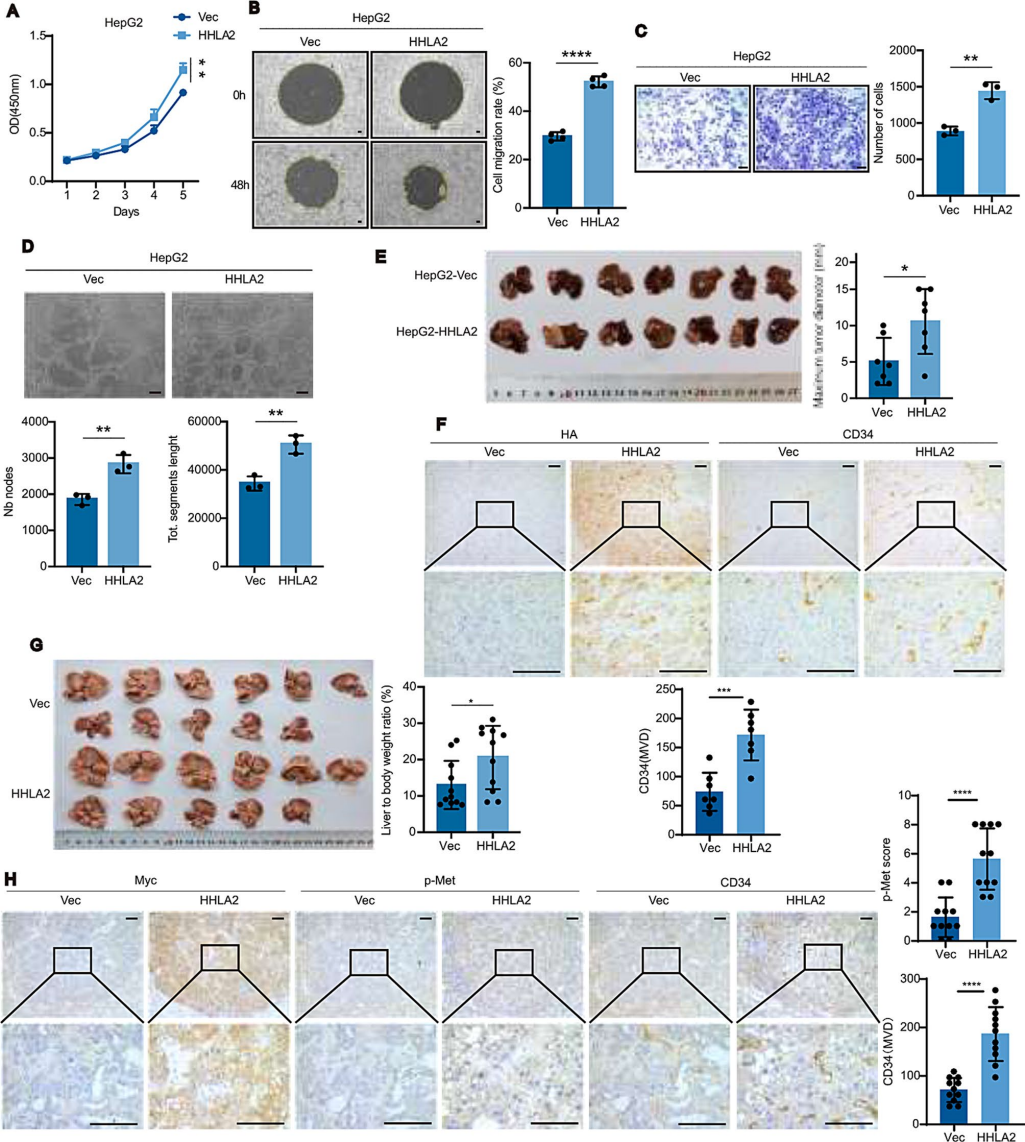
HHLA2 promotes aggressive phenotypes in HCC. (**A**–**D**) Assays for tumorigenic phenotypes of HepG2-Vec and HepG2-HHLA2 cells: (**A**) cell proliferation assessed by CCK-8 assay; (**B**) cell migration assay; (**C**) Transwell invasion assay; (**D**) tube formation assay in HUVECs using conditioned media (CM) from HepG2 cells. (**E**) Representative images of liver tumors from orthotopic xenograft models in nude mice injected with HepG2-Vec or HepG2-HHLA2 cells (*n* = 7 mice per group). (**F**) Representative immunohistochemical staining for CD34 (blood vessels) in sections from tumors shown in (**E**) (upper panel). Quantification of CD34 (Microvessel Density, MVD) is shown in the bottom panel. (**G**) Representative images of liver tumors (left) and quantification of tumor burden via liver/body weight ratio (right) in C57BL/6 mice (*n* = 11 per group) 4.5 weeks after HDTVi. Mice received plasmids encoding N-RasV12/myr-AKT1/Sleeping Beauty (SB) transposon system along with either a control vector or myc-HHLA2. (**H**) Representative immunohistochemical staining for CD34 (blood vessels), pY1235-Met (activated c-Met), and myc (HHLA2 marker) in sections from tumors shown in (**G**). Based on quantification of CD34 (MVD) and p-Met/myc (scoring) as described in Methods, increased c-Met activation and microvessel density were observed in HHLA2-overexpressing tumors compared to controls (bottom panel). *P* values were determined by one-way ANOVA (A) or two-tailed Student’s *t* test (**B**–**H**). * *P* < 0.05, ** *P* < 0.01, *** *P* < 0.001, **** *P* < 0.0001. Scale bars, 100 μm

To investigate HHLA2’s functional role, we utilized HCC cell lines exhibiting varying endogenous HHLA2 levels. HepG2 and Hep3B cells showed low HHLA2 expression, while Huh7 cells displayed high expression (Supplemental Fig. 2A, B). We generated stable cell lines that either overexpress HHLA2 (HepG2-HHLA2, Hep3B-HHLA2) or have HHLA2 knockdown by shRNA (Huh7-HHLA2KD) (Supplemental Fig. 2C, D). Overexpression of HHLA2 significantly promoted proliferation, colony formation, anchorage-independent growth, migration, and invasion in HepG2 and Hep3B cells (Fig. 1A-C, Supplemental Fig. 2F-K, 3B-E). Conversely, HHLA2 knockdown in Huh7 cells inhibited these pro-tumorigenic effects, which were restored upon HHLA2 reintroduction (Supplemental Fig. 2G-J, 3 A, C, E, G).

Furthermore, HHLA2 was found to promote tumor angiogenesis. Conditioned media from HHLA2-overexpressing HCC cells significantly enhanced human umbilical vein endothelial cell (HUVEC) tube formation (Fig. 1D, Supplemental Fig. 3F), whereas conditioned media from HHLA2-knockdown cells inhibited this process (Supplemental Fig. 3G). To evaluate in vivo effects, orthotopic xenografts of HepG2-HHLA2 cells grew larger tumors in nude mice compared to HepG2-Vec controls (Fig. 1E). Conversely, knockdown of HHLA2 via shRNA in Huh7 cells significantly reduced orthotopic tumor growth (Supplemental Fig. 3H). Immunohistochemistry (IHC) confirmed higher HHLA2 (HA-tag) expression and revealed increased CD34 + microvessel density, indicative of enhanced angiogenesis (Fig. 1F). Furthermore, to assess HHLA2’s impact in vivo, we utilized an established and rapid hydrodynamic tail vein injection (HDTVi) model in which oncogenic N-RasV12/myr-AKT1 activates key signaling pathways relevant to HCC and c-Met activity [28, 29]. Co-expression of myc-tagged HHLA2 in this model increased overall tumor burden, measured by liver-to-body weight ratio (Fig. 1G). Consistent with increased angiogenesis, these HDTVi tumors also showed higher CD34 + vessel density and elevated levels of phosphorylated c-Met (pY1235) via IHC (Fig. 1H). Collectively, our findings demonstrate that HHLA2 promotes HCC cell proliferation, migration, and invasion in vitro, and enhances tumor growth and angiogenesis in vivo.

### HHLA2 interacts with and activates c-Met, driving oncogenic signaling pathways

**Fig. 2:**
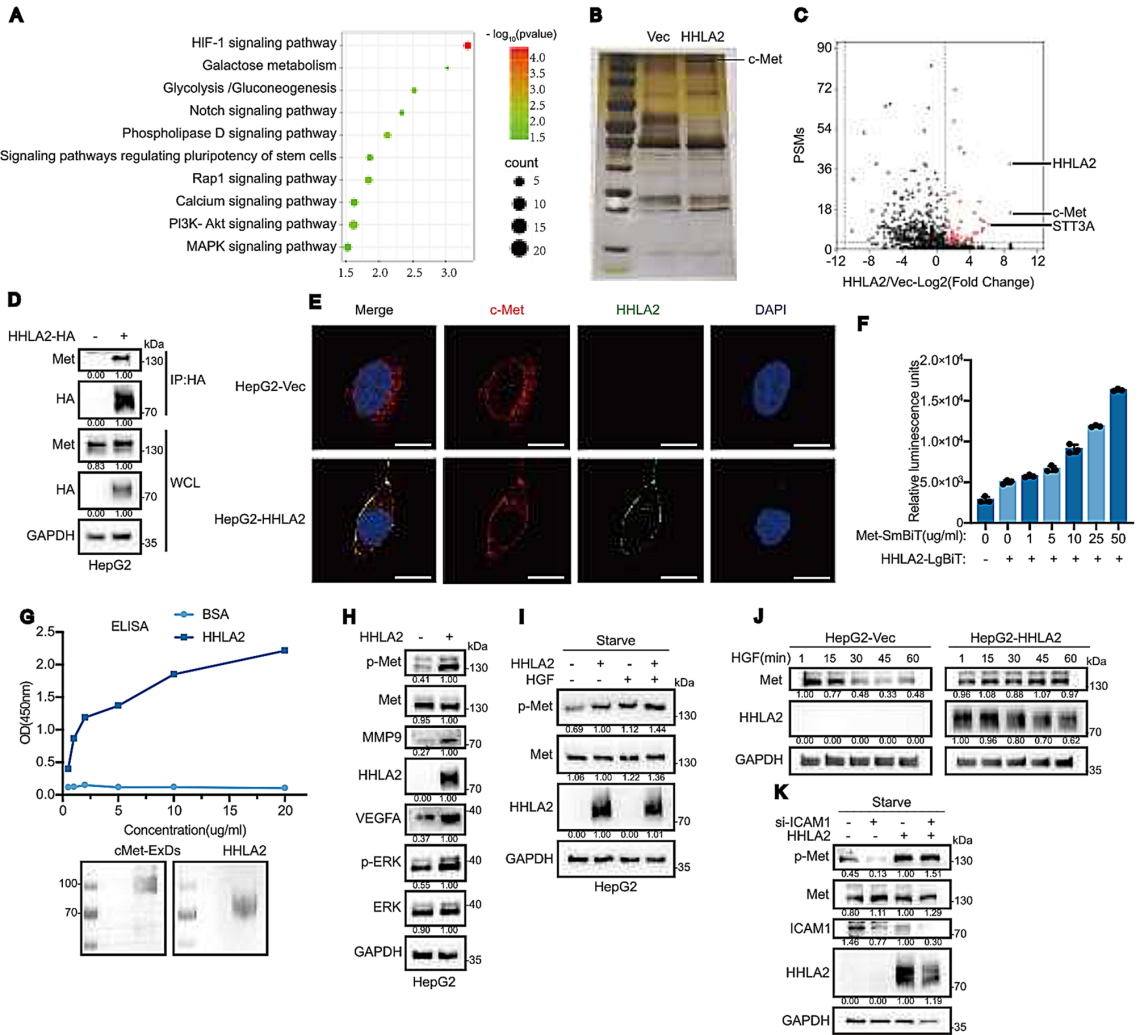
HHLA2 interacts with and constitutively activates c-Met. (**A**) KEGG pathway enrichment analysis of the HepG2 transcriptome after HHLA2 overexpression. (**B**) Silver-stained SDS-PAGE gel of anti-HA immunoprecipitates from HepG2-HHLA2-HA and HepG2-Vec cells. (**C**) Volcano plot depicting the differential abundance of proteins identified by mass spectrometry in HHLA2 versus vector immunoprecipitates from (**B**). The fold change in protein abundance (HHLA2/vector) is plotted on the *x* axis against peptide-spectrum match [PSM]) on the *y* axis. (**D**) Coimmunoprecipitation of HHLA2-HA and c-Met with anti-HA antibody in HepG2 cells with or without HHLA2 overexpression. (**E**) Representative immunofluorescence images of HHLA2 and c-Met in HepG2-Vec (upper panel) and HepG2-HHLA2 (lower panel) cells. Cells were serum starved overnight and then stimulated with 1 µM HGF for 15 min before fixation. Scale bar, 4 μm. (**F**) Split luciferase complementation assay to define the interaction domains between HHLA2 and c-Met. Luminescence was measured following the addition of indicated amounts of recombinant c-MET-ExD-SmBit-6His protein to 293-LgBiT-HHLA2 cells. (**G**) In vitro binding assay to examine the HHLA2 and c-Met interaction. Indicated amounts of HHLA2 ExD-SmBit-6His recombinant protein or BSA control were added to 96-well plates precoated with 1 µ g/well of purified c-MET-ExD-SmBit-6His protein and incubated for 1 h. Following washes, the amount of HHLA2 protein bound to c-Met was detected by ELISA. The lower panel shows the input purified c-MET-ExD-SmBit-6His and HHLA2 ExD-SmBit-6His recombinant proteins. (**H**,** I**) Western blot analysis of the indicated proteins in HepG2 cells with or without HHLA2 overexpression under normal conditions (H) or when cells were serum starved and then stimulated with 40 ng/ml HGF for 30 min (**I**). (**J**) Analysis of c-Met degradation following activation: HepG2-Vec or HepG2-HHLA2 cells were serum starved overnight and then stimulated with 40 ng/ml HGF for the indicated times. (**K**) Western blot analysis of the indicated proteins in HepG2-Vec or HepG2-HHLA2 cells following ICAM1 knockdown. All Western blots shown are representative of three independent experiments; relative quantification values are indicated below the bands, and full quantitative data/statistical analysis are provided in the supplementary Excel spreadsheet

To explore the underlying mechanisms of HHLA2-mediated HCC progression, we analyzed RNA-sequencing data from TCGA and protein expression data from The Cancer Proteome Atlas (TCPA). Elevated HHLA2 expression was found to be correlated with increased levels of p-Met, p-MEK1, eIF4E, and c-Myc, alongside decrease in the levels of p-AMPK and p-GSK3 (Supplemental Fig. 4A). Kyoto Encyclopedia of Genes and Genomes (KEGG) pathway analysis further demonstrated enrichment of genes in the PI3K-AKT and MAPK signaling pathways in HCC with high HHLA2 expression (Supplemental Fig. 4B). Transcriptome analysis of HHLA2-overexpressing HepG2 cells revealed significant alterations in gene expression (244 upregulated and 552 downregulated genes;|log2(fold change)| > 0.4, *p* < 0.05) (Supplemental Fig. 4C). KEGG analysis highlighted enrichment in PI3K-AKT and MAPK pathways (Fig. 2A), while GSEA showed enrichment in cell proliferation processes, including DNA endoreduplication and positive regulation of spindle checkpoint (Supplemental Fig. 4D).

To identify potential HHLA2-interacting proteins, we performed label-free, semi-quantitative mass spectrometry analysis of immunoprecipitated HHLA2 from HepG2-HHLA2 cells. This analysis identified c-Met and STT3 as prominent candidate interactors (Fig. 2B, C). Given c-Met’s established role in cellular processes and its activation of PI3K/AKT and MAPK pathways upon HGF binding, [30] which aligns with our transcriptomic enrichment analysis showing HHLA2 overexpression in HCC, we further investigated this interaction. Co-immunoprecipitation assays in HepG2 and Hep3B cells confirmed reciprocal interaction between HHLA2 and c-Met (Fig. 2D, Supplemental Fig. 5A, B), with confocal microscopy revealing their co-localization at the cell membrane (Fig. 2E). Using split-luciferase complementation assay, [31] we found that HEK293 cells expressing LgBiT-tagged c-Met showed dose-dependent increases in luminescent signal upon addition of recombinant SmBiT-tagged HHLA2-ExD (extracellular domain), indicating direct binding between their extracellular regions. Similar results emerged when HEK293 cells expressing LgBiT-tagged HHLA2 were incubated with c-Met-ExD harboring a SmBiT tag (Fig. 2F, Supplemental Fig. 5C). We further validated this interaction using purified ExDs of HHLA2 and c-Met from suspension-cultured 293f cells in an ELISA assay, which showed dose-dependent binding (Fig. 2G).

Investigation of HHLA2’s role in c-Met activation revealed that it functions as an agonist rather than a co-receptor. HHLA2 overexpression increased c-Met phosphorylation at Y1235, activated downstream Erk, and upregulated MMP9 and VEGFA (Fig. 2H, Supplemental Fig. 5G). Notably, even under serum starvation, c-Met phosphorylation persisted in HHLA2-expressing cells but was abolished in controls (Fig. 2I). These findings were validated in Huh7 cells, where HHLA2 knockdown reduced phosphorylated c-Met, phosphorylated Erk, MMP9, and VEGFA levels, with effects reversed upon HHLA2 replenishment (Supplemental Fig. 5H, I).

HHLA2-mediated c-Met activation differed from HGF stimulation in several aspects. While HGF triggered c-Met internalization and gradual degradation,^32^ HHLA2 maintained c-Met’s membrane localization and prevented its degradation, leading to sustained signaling in HepG2 cells (Fig. 2E, F, J). Furthermore, HHLA2 could functionally substitute for ICAM-1, a known c-Met co-receptor in HepG2 cells [33], maintaining c-Met phosphorylation even under serum-starved conditions where ICAM-1 knockdown abolished HGF-induced activation (Fig. 2K).

Given that HHLA2 undergoes glycosylation by the STT3 oligosaccharyltransferase complex in colorectal cancer [34], and that STT3A was identified as an HHLA2-interacting protein in our study (Fig. 2C), we investigated the role of HHLA2 glycosylation in c-Met binding. Treatment of Tunicamycin, an N-linked glycosylation inhibitor, abolished HHLA2 glycosylation and reduced c-Met binding in HepG2 cells (Supplemental Fig. 5D). This was confirmed using un-glycosylated recombinant HHLA2-SmBiT from Tunicamycin-treated 293f cells, which failed to interact with c-Met in split-luciferase assays, while glycosylated HHLA2-SmBiT readily did so (Supplemental Fig. 5E, F).

In summary, HHLA2 interacts with c-Met through its extracellular domains, leading to ligand-independent c-Met activation and stabilization at the cell membrane. This interaction triggers downstream signaling pathways, notably the MAPK pathway, contributing to HCC progression. This direct activation of c-Met by HHLA2 represents a novel mechanism by which HHLA2 promotes tumor growth and aggressiveness.

### HHLA2-mediated HCC progression and metastasis are dependent on c-Met activation and reversible by c-Met Inhibition

**Fig. 3:**
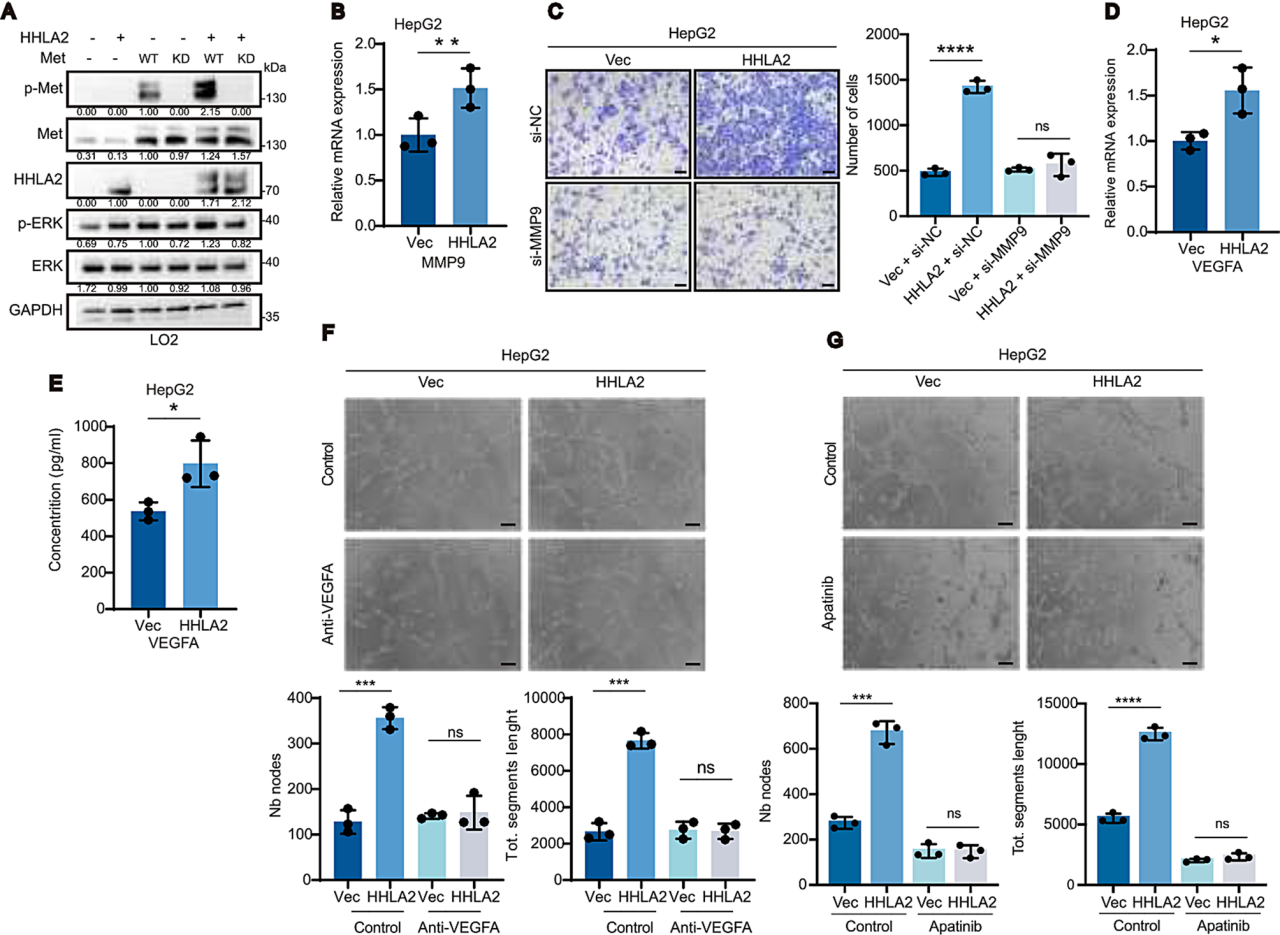
MMP9 and VEGFA mediate HHLA2-promoted invasion and tumor angiogenesis, respectively. (**A**) Western blot analysis of the indicated proteins in LO2 cells cotransfected with HHLA2 and c-MET (wild-type or kinase-dead) in the combinations shown. Western blots shown are representative of three independent experiments; relative quantification values are indicated below the bands, and full quantitative data/statistical analysis are provided in the supplementary Excel spreadsheet. (**B**) qRT-PCR analysis of *MMP9* mRNA expression in HepG2 cells with or without HHLA2 expression. (**C**) Transwell invasion assays of HepG2-Vec and HepG2-HHLA2 cells with or without MMP9 knockdown. (**D**,** E**) *VEGFA* mRNA expression assessed by qRT-PCR (**D**) and VEGFA secretion assessed by ELISA (**E**) in HepG2-Vec and HepG2-HHLA2 cells. (**F**,** G**) HUVEC tube formation assays using conditioned media from HepG2-Vec or HepG2-HHLA2 cells, with or without VEGFA depletion using an anti-VEGFA antibody (**F**) or in the presence or absence of 50 ng/ml apatinib (VEGF receptor inhibitor) (**G**). *P* values were determined by two-tailed Student’s *t* test. * *P* < 0.05, ** *P* < 0.01, *** *P* < 0.001, **** *P* < 0.0001. Scale bars, 100 μm

**Fig. 4:**
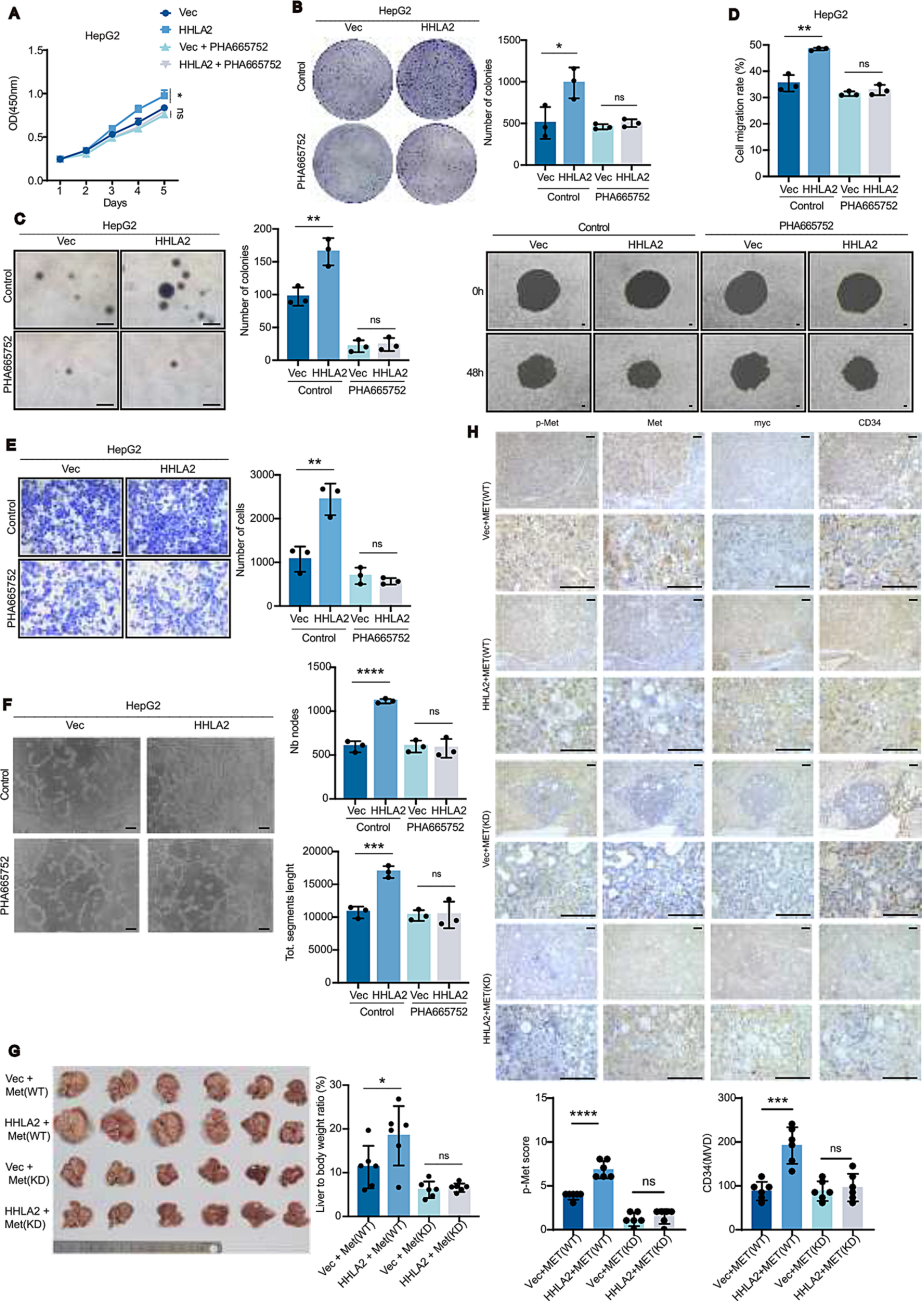
c-Met activation is indispensable for HHLA2-promoted HCC progression. (**A**–**F**) Assessment of malignant phenotypes in HepG2-Vec and HepG2-HHLA2 cells, with or without 1µM c-Met inhibitor PHA665752 treatment. Phenotypes evaluated include (**A**) cell proliferation by CCK-8 assay, (**B**) two-dimensional colony formation, (**C**) soft agar colony formation, (**D**) cell migration, (**E**) Transwell invasion, and (**F**) HUVEC tube formation with conditioned media. (**G**) Representative images of orthotopic liver tumors and quantification of tumor burden (liver/body weight ratio) in C57BL/6 mice following HDTVi of plasmids encoding myc-HHLA2 or a control vector, along with plasmids encoding either wild-type c-MET or kinase-dead c-MET, and the myr-AKT1 + N-RasV12 + SB (*n* = 6 mice per group). Due to the diffuse nature of tumor growth in this model, liver/body weight ratio was used as the primary measure of tumor burden. (**H**) Immunohistochemical staining and quantification of tumor tissues from **G** for phosphorylated c-Met (p-Met; pY1235, indicative of activated c-Met), total c-Met, myc (HHLA2 marker), and CD34 (blood vessel marker). IHC staining was quantified as described in the Methods; representative images are shown. *P* values were determined by one-way ANOVA (**A**) or two-tailed Student’s *t* test (**B**–**H**). * *P* < 0.05, ** *P* < 0.01, *** *P* < 0.001, **** *P* < 0.0001. Scale bars, 100 μm

The oncogenic effects of HHLA2 are primarily mediated through c-Met-dependent signaling cascades. HHLA2 overexpression enhanced Erk phosphorylation in both HepG2 and Hep3B cells, while its knockdown in Huh7 cells attenuated Erk activation - an effect that could be rescued by HHLA2 re-expression (Fig. 2H, Supplemental Fig. 5G-I). To definitively establish the requirement for functional c-Met in HHLA2-mediated Erk activation, we utilized the non-tumorigenic LO2 hepatocyte cell line, which expresses deficient levels of endogenous c-Met. While c-Met overexpression alone in LO2 cells induced its autophosphorylation and activated Erk, co-expression of c-Met with HHLA2 further enhanced the phosphorylation of both proteins. Critically, expression of the kinase-deficient c-Met KD mutant (Y_1234,1235_F), [35] either alone or in combination with HHLA2, failed to induce Erk phosphorylation in LO2 cells (Fig. 3A). This finding, together with the observation that c-Met KD mutant abrogates HHLA2-driven tumor promotion in HDTVi HCC mouse models (Fig. 4G), strongly supports the necessity of functional c-Met and its kinase activity for HHLA2-dependent Erk activation. Furthermore, it demonstrates the dominant-negative effect of c-Met KD mutant in blocking endogenous c-Met activation.

To identify the c-Met signaling effector responsible for HHLA2-promoted HCC metastasis (metastasis and angiogenesis being the most prominent HHLA2-induced phenotypes), we examined the effect of HHLA2 overexpression in HepG2 cells. We found no evidence of epithelial-mesenchymal transition (EMT), as E-cadherin and N-cadherin expression remained unchanged, and no morphological alterations were observed (Supplemental Fig. 6A-C). While HHLA2 did not affect MMP2 and MMP7 expression, it significantly upregulated MMP9 (Fig. 3B, Supplemental Fig. 6D, H), and gelatin zymography confirmed a corresponding increase in MMP9 enzymatic activity in conditioned media (Supplemental Fig. 6J). Conversely, HHLA2 knockdown in Huh7 cells suppressed MMP9 expression, which could be rescued by HHLA2 re-expression (Supplemental Fig. 6I). The functional significance of MMP9 was demonstrated through siRNA-mediated knockdown in HepG2 cells, which effectively abolished HHLA2 overexpression-induced invasion, while MMP2 knockdown had no impact (Fig. 3C, Supplemental Fig. 6-G, K). Similarly, MMP9 knockdown reduced Huh7 cell invasiveness but did not further impair invasion in Huh7-HHLA2KD cells (Supplemental Fig. 6L).

We also found that VEGFA, downstream of c-Met signaling, is responsible for the pro-angiogenic function of HHLA2 in HCC. RNA-seq analysis revealed enrichment of VEGF signaling genes (VEGFA, PTGS2, PLCG2) upon HHLA2 overexpression in HepG2 cells (Supplemental Fig. 4C). Stable HHLA2 overexpression in HepG2 and Hep3B cells increased VEGFA mRNA levels, protein abundance, and secretion, while HHLA2 knockdown in Huh7 cells showed opposite effects, restorable by HHLA2 re-expression (Figs. 2H and 3D and E; Supplemental Fig. 5G, H, 6 M-P). In vitro tube formation assays demonstrated that VEGFA depletion from HepG2-conditioned media using a specific antibody significantly attenuated HHLA2’s pro-angiogenic effects (Fig. 3F). Similarly, VEGFR2 inhibition with apatinib abolished HHLA2-enhanced tube formation (Fig. 3G, Supplemental Fig. 6Q).

The therapeutic potential of targeting c-Met was extensively validated in vitro. The c-Met inhibitor PHA665752 effectively blocked HHLA2-induced Erk activation, VEGFA and MMP9 upregulation in HepG2, Hep3B, and Huh7 cells (Supplemental Fig. 7A-D, F-H). Furthermore, HHLA2-mediated enhancement of proliferation, 2-D and 3-D colony formation, migration, invasion, and angiogenesis was significantly suppressed by PHA665752 treatment (Fig. 4A-F, Supplemental Fig. 8A-I). We further tested the clinically relevant multi-kinase inhibitors cabozantinib on HepG2-Vec and HepG2-HHLA2 cells. Consistent with PHA665752 data, HHLA2 overexpression significantly increased sensitivity to cabozantinib (Supplemental Fig. 9), suggesting HHLA2 may predict responsiveness to multi-kinase inhibitors targeting c-Met. siRNA-mediated c-Met knockdown produced similar effects (Supplemental Fig. 7E, I, J, Supplemental Fig. 10A-F).

To confirm c-Met’s in vivo role in HHLA2-driven tumorigenesis, we used the HDTVi HCC mouse model. Co-injection either wild-type (WT) or kinase-dead (KD) c-Met, along with HHLA2 or control vector, demonstrated HHLA2 enhanced tumor growth in WT c-Met mice but not in KD c-Met mice (Fig. 4G). Immunohistochemistry showed increased c-Met phosphorylation and CD34 + microvessel density in HHLA2-overexpressing tumors with WT c-Met, but not with KD c-Met (Fig. 4H).

**Fig. 5:**
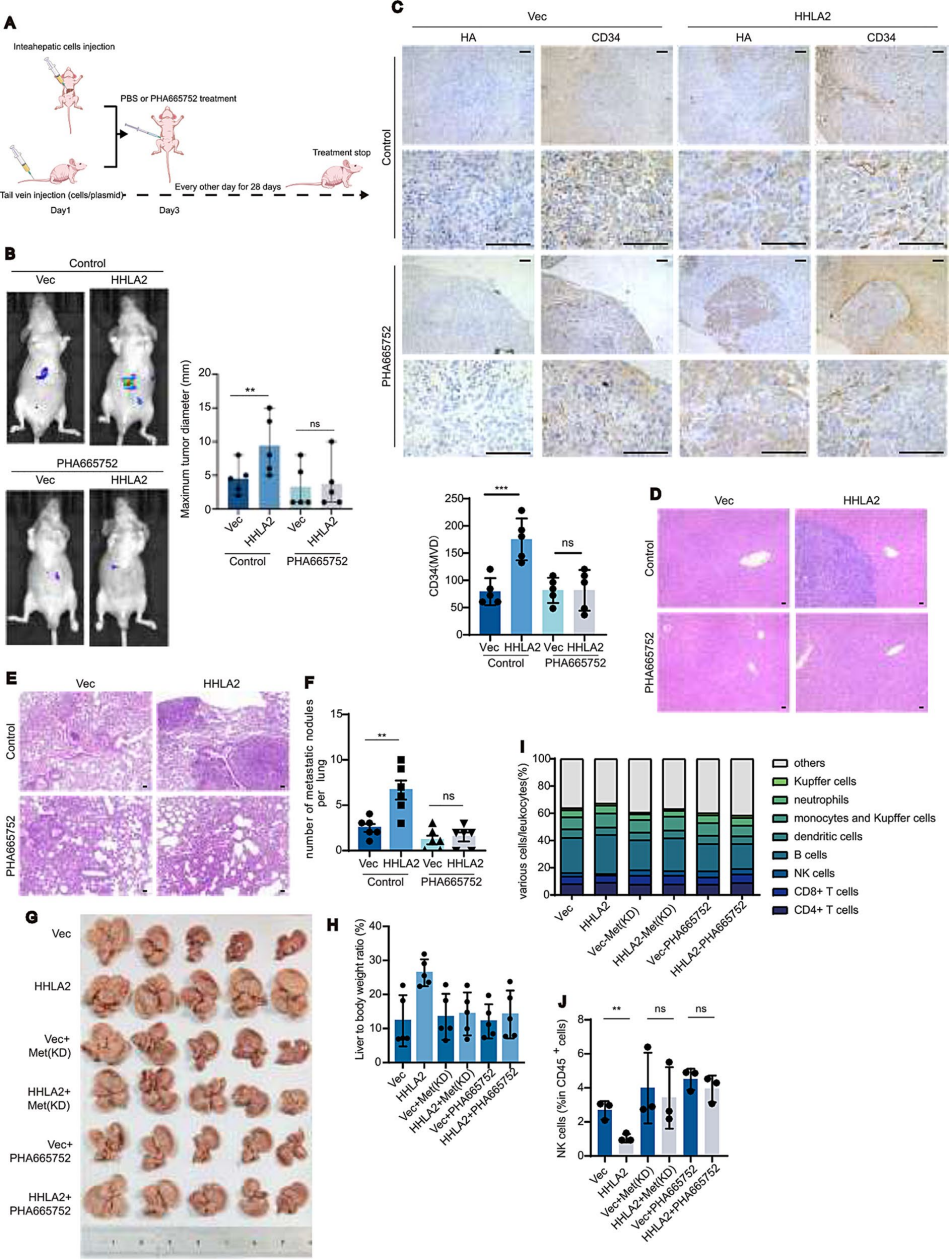
c-Met inhibition abrogates HHLA2-driven HCC progression. (**A**) Schematic representation of intraperitoneal PHA665752 administration. Dosing began on day 3 after HDTVi and continued every other day for 28 days at 20 mg/kg per dose. (**B**) Representative images and quantification of orthotopic HCC xenografts in nude mice. Mice were injected with HepG2-Vec or HepG2-HHLA2 cells, followed by intraperitoneal injection of PHA665752 as outlined in A. The left panel shows representative images of the mice, and the right panel quantifies the largest tumor size (*n* = 5 mice per group). (**C**) Immunohistochemical staining of xenograft livers for HA (HHLA2) and CD34 (blood vessels). Quantification of CD34 is shown in the bottom panel. Tissues were obtained from the experiment described in (**B**). (**D**) H&E staining of uninjected liver lobes to assess HCC metastasis. HepG2-Vec or HepG2-HHLA2 cells were injected into the left liver lobe, followed by PHA665752 treatment as described in (**A**). (**E**,** F**) Assessment of HCC lung metastasis in nude mice following tail vein injection of HepG2-Vec or HepG2-HHLA2 cells and PHA665752 treatment. (**E**) H&E staining of lung tissues. (**F**) Quantification of tumor nodules. PHA665752 treatment was administered as described in (**A**) (*n* = 6 mice per group). (**G–J**) Orthotopic liver tumor development and immune cell infiltration in C57BL/6 mice. (**G**) Representative images of orthotopic liver tumors following HDTVi of myc-HHLA2 or a control vector, along with N-RasV12 + myr-AKT1 + SB. (**H**) Quantification of tumor burden (liver/body weight ratio). Due to the diffuse nature of tumor growth in this model, liver/body weight ratio was used as the primary measure of tumor burden. (**I**) Overall proportion of immune cells in different liver tissues. (**J**) Quantification of NK cell abundance. In this model, either kinase-dead MET (MET-KD) was delivered via HDTVi or PHA665752 was administered as described in **A**. Data are presented as mean ± SD (*n* = 5 mice per group). *P* values were determined by two-tailed Student’s *t* test. * *P* < 0.05, ** *P* < 0.01, *** *P* < 0.001, **** *P* < 0.0001. Scale bars, 100 μm

The impact of pharmacological c-Met inhibition on HHLA2-driven HCC progression and metastasis was evaluated using orthotopic and tail vein xenograft models. In both models, mice were treated with PHA665752 every other day, starting on day three post-injection (Fig. 5A). In orthotopic xenograft models, HepG2-HHLA2 cells exhibited accelerated intrahepatic tumor growth and increased CD34 + microvascular density compared to HepG2-Vec controls (Fig. 5B, C, Supplemental Fig. 11A). Treatment with PHA665752 effectively countered these effects and prevented intrahepatic metastasis to un-injected liver lobes (Fig. 5A, B, Supplemental Table 3). In the tail vein injection model, HHLA2-overexpressing cells formed significantly more lung metastatic nodules, an effect also suppressed by PHA665752 treatment (Fig. 5E, F, Supplemental Fig. 11B). Consistent results were observed in the myr-AKT1 + N-RasV12-driven HDTVi mouse model of HCC (Fig. 5G, H). HHLA2 overexpression significantly increased hepatic tumor load in these mice (Fig. 5G). However, this increase was completely abrogated when endogenous c-Met was inhibited, either pharmacologically with PHA665752 or genetically through expression of dominant-negative kinase-dead (KD) c-Met (Fig. 5G). Furthermore, PHA665752 treatment in KD c-Met mice provided no additional benefit, reinforcing the conclusion that HHLA2-mediated activation of c-Met is critical for driving HCC progression.

Although HHLA2 is known for its immunosuppressive function in HCC,^27^ we aimed to differentiate its oncogenic contribution to HCC progression. Therefore, we examined the immunological impact of HHLA2 within the myr-AKT1 + N-RasV12-driven HDTVi model. Flow cytometry analysis revealed that neither HHLA2 overexpression nor c-Met inhibition (via dominant-negative mutant or PHA665752) significantly affected infiltration or activity of most immune cell populations (B cells, monocytes, dendritic cells, neutrophils, Kupffer cells, CD4 + and CD8 + T cells) (Fig. 5I, Supplemental Fig. 11C-E). However, HHLA2 specifically suppressed NK cell infiltration, an effect that was completely reversed by either KD c-Met expression or PHA665752 treatment (Fig. 5J), suggesting this suppression was mediated through hepatocyte c-Met signaling rather than direct effects on NK cells.

In conclusion, HHLA2-driven HCC progression is critically dependent on functional c-Met signaling and its downstream pathways, as demonstrated through comprehensive genetic and pharmacological approaches. The therapeutic efficacy of c-Met inhibition in blocking HHLA2-mediated tumor progression suggests a promising treatment strategy for HHLA2-positive HCC.

### HHLA2 drive c-Met activation and predicts c-Met inhibitor response in patient samples and organoids

**Fig. 6:**
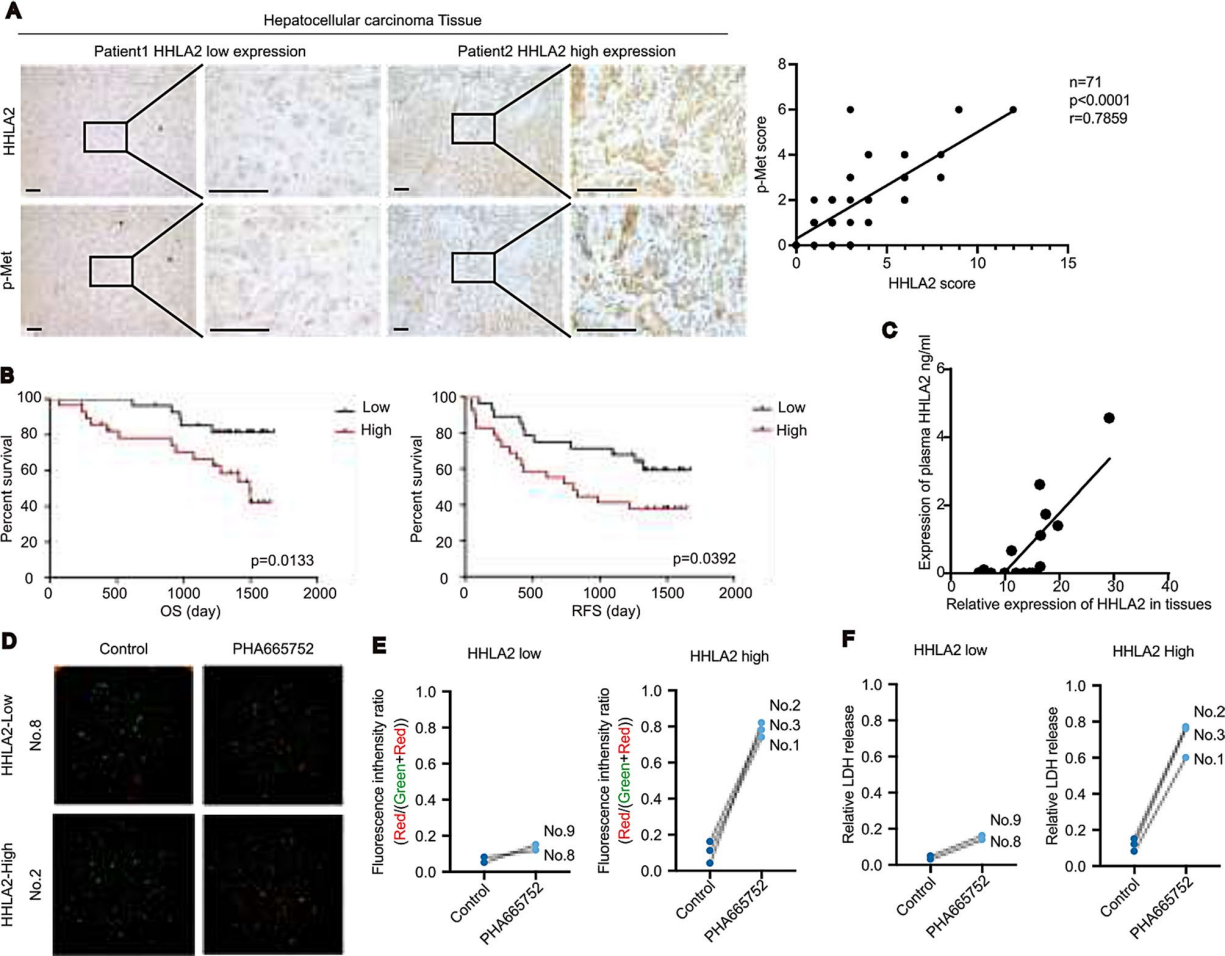
HHLA2 expression correlates with c-Met phosphorylation and predicts efficacy of c-Met inhibitor therapy in HCC. (**A**) Representative images and quantification of p-MET (pY1235) and HHLA2 expression in 71 HCC tissues. Immunohistochemical staining assessed p-MET and HHLA2 expression (left panel). The correlation between p-MET and HHLA2 expression levels was quantified (right panel). Scoring methods are detailed in the Methods section. *P* value was determined by Pearson correlation analysis. *P* < 0.0001. (**B**) Kaplan-Meier analysis of OS and recurrence-free survival (RFS) in patients with HCC with high and low HHLA2 expression from the cohort described in (**A**). *P* < 0.05. (**C**) Correlation between *HHLA2* mRNA expression in HCC tissues and corresponding serum HHLA2 protein levels. *x* axis: *HHLA2* mRNA expression in HCC tissues quantified by qRT-PCR; *y* axis: serum HHLA2 protein levels quantified by ELISA. (**D**–**F**) Patient-derived organoid (PDO) response to c-Met inhibition. PDOs derived from HCC tissues in (**A**), with two exhibiting low HHLA2 expression and three exhibiting high HHLA2 expression, were treated with 15 µM PHA665752 or DMSO for 48 h. (**D**) Representative images showing PDO death. (**E**) Quantification of PDO death. Dead cells are stained red with propidium iodide (PI), and live cells are stained green with Calcein-AM. (**F**) LDH levels in PDO culture supernatants as a measure of cytotoxicity. Scale bars, 100 μm

In a study of 71 HCC tumor samples from Shanghai, China, we found a strong positive correlation between HHLA2 expression and c-Met phosphorylation (Fig. 6A). Specifically, tumors with high HHLA2 levels were significantly more likely to exhibit strong c-Met activation than tumors with low HHLA2 levels (Supplemental Table 4). Patients with high HHLA2 expression showed significantly shorter overall and progression-free survival (Fig. 6B). This corroborates findings in the earlier cohort of 176 patients in our study and further suggest that HHLA2 promotes HCC progression, likely via c-Met activation.

We explored the potential of HHLA2 as a liquid biopsy marker, similar to other B7 family members like PD-L1 and B7-H3 [36, 37]. While standard methods failed to detect HHLA2 in HCC cell line supernatants, we successfully detected it in the serum of 13 patients with high tumor HHLA2 expression. Notably, serum HHLA2 levels correlated positively with tumor HHLA2 levels (Fig. 6C), suggesting its potential as a non-invasive biomarker, although more sensitive detection methods are needed.

To investigate if HHLA2 predicts c-Met inhibitor response, we analyzed data from the Cancer Cell Line Encyclopedia (CCLE). While neither c-Met protein levels (RPPA) nor HHLA2 mRNA expression alone correlated with c-Met inhibitor efficacy (Supplemental Fig. 12A, 12B), in cell lines with aberrant c-Met expression, higher HHLA2 mRNA levels were associated with increased sensitivity to c-Met inhibitors (Supplemental Fig. 12C). We validated this finding using patient-derived organoids (PDOs) from five HCC patients with similar c-Met expression (Supplemental Fig. 12D, E). PDOs with high HHLA2 expression showed significantly greater cell death and cytotoxicity in response to the c-Met inhibitor PHA665752 compared to those with low HHLA2 expression (Fig. 6D-F, Supplemental Fig. 12F), suggesting HHLA2 can predict response to c-Met inhibitors in HCC.

In summary, our study reveals that HHLA2 enhances c-Met phosphorylation, driving HCC progression, and demonstrates that HHLA2 levels, detectable in patient serum, can potentially serve as a liquid biopsy marker to predict the efficacy of c-Met inhibitor treatment in HCC.

## Discussion

Here, we report an oncogenic function of HHLA2 in HCC progression, establishing its direct interaction with and subsequent constitutive activation of c-Met as a crucial driver of HCC development and metastasis. These findings challenge the prevailing understanding of HHLA2 as solely an immune checkpoint molecule, [38] highlighting its ability to modulate oncogenic signals independent of its known immunosuppressive functions directly. Our work positions HHLA2 as a potential therapeutic target and, crucially, a potential non-invasive prognostic biomarker for predicting c-Met inhibitor efficacy in HCC, paving the way for precision therapy in HCC.

While HHLA2’s oncogenic potential has been suggested in other cancers, such as lung and gastric cancer, [39–41] the underlying mechanisms remained unclear. Here, we provide compelling evidence of a direct interaction between HHLA2 and c-Met’s extracellular domains, leading to constitutive c-Met activation independent of its ligand, HGF. This activation deviates significantly from the canonical HGF-induced pathway, which typically involves c-Met internalization and transient signaling. HHLA2, conversely, stabilizes c-Met at the cell membrane, sustaining downstream signaling and promoting continuous proliferation, invasion, and angiogenesis. Furthermore, HHLA2 bypasses the need for the c-Met co-receptor ICAM-1, suggesting an alternative activation mechanism with potential implications for tumors lacking ICAM-1 expression. Our findings also highlight the crucial role of HHLA2 N-glycosylation in facilitating c-Met binding and activation, offering a potential avenue for therapeutic intervention by targeting this modification.

Mechanistically, we identified MMP9 and VEGFA as key downstream effectors of HHLA2-mediated c-Met activation, driving HCC invasion and angiogenesis, respectively. Inhibiting either MMP9 or VEGFA signaling effectively counteracts HHLA2’s pro-tumorigenic effects, underscoring their importance in HCC progression. Both in vitro and in vivo studies confirmed that c-Met activation is essential for HHLA2-driven oncogenesis, with c-Met inhibition effectively abolishing HHLA2’s tumor-promoting activities.

Our findings in the HDTVi HCC mouse model suggest that HHLA2’s primary oncogenic contribution is through direct tumor promotion rather than generalized immune suppression. The absence of HHLA2’s receptors, TMIGD2 and KIR3DL3, [42, 43] on murine immune cells may explain the lack of broad immunosuppression observed across most studied immune populations. However, we did observe a specific suppression of NK cell infiltration by HHLA2. Importantly, this effect was reversed by c-Met inhibition (Fig. 5J), pointing strongly towards an indirect, c-Met-dependent immunosuppressive mechanism likely originating from signaling changes within the hepatocytes. While c-Met is also expressed on immune cells, [44] potentially allowing for direct HHLA2 interaction, our in vivo results favour the indirect pathway as the dominant effect in this model, given the specific nature of the NK cell suppression and lack of broader immune changes. A limitation is that we did not directly assess HHLA2’s impact on immune cell c-Met; therefore, further investigation is needed to fully elucidate the interplay between HHLA2, c-Met signaling on both tumor and immune cells, and the overall immune microenvironment.

The strong clinical correlation between HHLA2 expression and c-Met phosphorylation in patient samples, along with the association of high HHLA2 levels with poor patient outcomes, reinforces HHLA2’s potential as a prognostic biomarker. Our detection of HHLA2 in the serum of HCC patients with high tumor HHLA2 expression underscores its potential as a non-invasive liquid biopsy marker for disease monitoring and identifying patients most likely to benefit from c-Met inhibitor therapy, offering a novel approach to precision medicine. Realizing this potential will require further assay development and validation, potentially employing high-sensitivity platforms such as digital ELISA (e.g., Simoa) or electrochemiluminescence (ECL) assays to ensure robust quantification in patient serum.

Our data strongly suggest that HHLA2 expression can predict c-Met inhibitor efficacy. The correlation between high HHLA2 expression and increased sensitivity in HCC cell lines and patient-derived organoids underscores its value in identifying tumors exhibiting ‘c-Met addiction’. Detecting HHLA2 in serum further highlights its potential as a non-invasive liquid biopsy marker, offering a promising avenue for personalizing c-Met inhibitor therapy and improving treatment outcomes. However, rigorous clinical validation is essential to translate the use of HHLA2, whether assessed in tissue or serum, into a reliable biomarker for patient stratification. Beyond predicting initial sensitivity, the potential role of HHLA2 in acquired resistance to RTK inhibitors also warrants investigation. Conceivably, tumors could evade therapy by upregulating HHLA2 expression or relying on its alternative, ligand-independent activation of c-Met. Future studies analyzing longitudinal patient samples during RTKI therapy or utilizing experimentally derived resistant models are needed to clarify HHLA2’s role in therapeutic resistance.

This study has limitations, including the use of only male mice and current methods’ limited sensitivity for detecting serum HHLA2, necessitating further assay development. Our in vivo HCC model, lacking the mouse HHLA2 receptor, could not fully elucidate HHLA2’s immune interactions. Future studies should investigate HHLA2’s effects on receptor-expressing immune cells and explore combining c-Met and immune checkpoint inhibitors in HHLA2-positive HCC. Although the CMV promoter utilized in our HDTVi model may have limitations in hepatocytes, significant HHLA2-driven phenotypic effects were observed; nonetheless, liver-specific promoters could be employed in future work. Structural analysis of the HHLA2/c-Met interaction could inform targeted therapy development. Despite these limitations, our findings strongly support HHLA2’s oncogenic role in HCC via c-Met activation, highlighting its potential as a therapeutic target and liquid biopsy biomarker.

## Electronic supplementary material

Below is the link to the electronic supplementary material.

Supplementary Material 1

## Data Availability

No datasets were generated or analysed during the current study.

## Abbreviations

MAPK: Mitogen-Activated Protein Kinase
PI3K: Phosphoinositide 3-Kinase
Akt: Protein Kinase B
STAT: Signal Transducer and Activator of Transcription
TCGA: The Cancer Genome Atlas
OS: Overall Survival
BCLC: Barcelona Clinic Liver Cancer
AFP: Alpha-Fetoprotein
CM: Conditioned Media
HUVECs: Human Umbilical Vein Endothelial Cells
HDTVi: Hydrodynamic Tail Vein Injection
TCPA: The Cancer Proteome Atlas
KEGG: Kyoto Encyclopedia of Genes and Genomes
GSEA: Gene Set Enrichment Analysis
ExD: Extracellular Domain
EMT: Epithelial-Mesenchymal Transition
MMP: Matrix Metalloproteinase
VEGFR: Vascular Endothelial Growth Factor Receptor
WT: Wild-Type
KD: Kinase-Dead
PDOs: Patient-Derived Tumor Organoids
CCLE: Cancer Cell Line Encyclopedia
RPPA: Reverse Phase Protein Array
HPA: Human Protein Atlas
RTK: Receptor tyrosine kinase
NK: Natural Killer
