# Supplementary material for: HHLA2 activates c-Met and identifies patients for targeted therapy in hepatocellular carcinoma
Source: J Exp Clin Cancer Res. 2025 May 20;44:153. doi: 10.1186/s13046-025-03407-6 (PMC12090556; doi:10.1186/s13046-025-03407-6)
Supplement: Supplementary file 1 — Supplementary Material 1 [file 13046_2025_3407_MOESM1_ESM.pdf]

Supplemental Methods

Antibodies and Reagents

The details of antibodies and reagents used are listed in the following table.

Antibodies and Drugs	Suppliers	Catalog Number	Clonality	Host Species	Concentrations/ dilutions
HHLA2/B7-H7	CST	52200S	Monoclonal	Rabbit	WB 1：1000
HHLA2	Abcam	ab214327	Polyclonal	Rabbit	IHC 1：100
c-Met	Proteintech	25869-1-AP	Polyclonal	Rabbit	WB 1：1000<br>IHC 1:500
p-Met(Tyr1234/1235)	CST	3077S	Monoclonal	Rabbit	WB 1：1000<br>IHC 1:100
HA-Tag	CST	3724S	Monoclonal	Rabbit	WB 1：1000<br>IHC 1:800
GAPDH	Proteintech	HRP-60004	Monoclonal	Mouse	WB 1：10000
VEGFA	Proteintech	19003-1-AP	Polyclonal	Rabbit	WB 1：2000
p44/42 MAPK (Erk1/2)	CST	4695S	Monoclonal	Rabbit	WB 1：1000
p-p44/42 MAPK(Erk1/2)	CST	4370S	Monoclonal	Rabbit	WB 1：2000
MMP9 (N-Terminal)	Proteintech	10375-2-AP	Polyclonal	Rabbit	WB 1：500
ICAM-1	Proteintech	10831-1-AP	Polyclonal	Rabbit	WB 1：5000
HGF β	CST	52445t	Monoclonal	Rabbit	WB 1：1000
human N-cadherin	Proteintech	22018-1-AP	Polyclonal	Rabbit	WB 1：2000
E-cadherin	Proteintech	60335-1-Ig	Monoclonal	Mouse	WB 1：2000
Myc	CST	2276S	Monoclonal	Mouse	IHC 1:500
CD34	ABclonal	A19015	Monoclonal	Rabbit	IHC 1:100
PHA665752	Targetmol	T6128			In vitro 1 μM<br>In vivo 20 mg/kg<br>Organoid 15μM
Cabozantinib	Yeasen	52724ES08			10μM
Tunicamycin	Beyotime	SC0393-10mM			1 μg/ml
Apatinib	Beyotime	SF5454-10mM			1 μM

Omics data analysis

HHLA2 expression and patient prognosis: Publicly available datasets were used to explore HHLA2 expression and its clinical relevance.

- HHLA2 protein expression data in normal and tumor tissues were obtained from the Human Protein Atlas (HPA)[46].
- To investigate the association between HHLA2 expression and patient prognosis, we analyzed gene expression and corresponding clinical follow-up data from The Cancer Genome Atlas (TCGA) [The Cancer Genome Atlas: https://portal.gdc.cancer.gov/].
- We further evaluated the relationship between HHLA2 expression or protein phosphorylation levels from The Cancer Proteome Atlas (TCPA) Reverse Phase Protein Array (RPPA) data [The CancerProteome Atlas: https://tcpaportal.org/tcpa/index.html)].

HHLA2 expression and drug sensitivity: The potential link between HHLA2 expression and cancer cell response to therapy was explored using data from the Cancer Cell Line Encyclopedia (CCLE) [http://www.broadinstitute.org/ccle].

- Gene expression (CCLE_DepMap_18Q4_RNAseq_log2 [TPM + 1]), protein expression (CCLE_RPPA_20180123.csv), and drug sensitivity (CCLE_NP24.2009_Drug_-data_2015.02.24) data were downloaded from the CCLE portal.
- Specifically, we analyzed the correlation between HHLA2 and c-Met expression levels with the half-maximal inhibitory concentration (IC50) of c-Met inhibitors.

Clinical samples

This study utilized two independent cohorts of human HCC tissues. The first cohort comprised 176 HCC samples and was employed to analyze HHLA2 expression and assess its correlation with clinicopathological features. Tissue specimens, including matched tumor and adjacent non-tumor tissues, were obtained from the Affiliated Cancer Hospital and Institute of Guangzhou Medical University. The study protocol was approved by the institution's Ethics Committee above, and written informed consent was obtained from all patients per the Declaration of Helsinki. The second cohort, encompassing 71 HCC samples, was used to evaluate the correlation between HHLA2 expression and p-Met levels. Tissue samples were procured from the Eastern Hepatobiliary Surgery Hospital in Shanghai, China. Approval for this study was granted

by the Ethics Committees of both the Affiliated Cancer Hospital of Guangzhou Medical University and the Eastern Hepatobiliary Surgery Hospital, with written informed consent obtained from all participants. All experiments and analyses were conducted with each patient's understanding and written consent.

Cell culture

Human HCC cell lines SK-Hep-1, Hep3B, Huh7, MHCC97H, HepG2, SMMC7721, and BEL7402 were obtained from the Cell Bank of Guangzhou Medical University of China. All cell lines were authenticated by short tandem repeat (STR) profiling within 6 months prior to use and routinely tested negative for mycoplasma contamination. Human HCC cell lines SK-Hep-1, Hep3B, Huh7, MHCC97H, and HepG2 were cultured in Dulbecco's Modified Eagle Medium (DMEM) supplemented with 10% fetal bovine serum (FBS). SK-Hep-1, SMMC7721 and BEL7402 cells were maintained in RPMI 1640 medium containing 10% FBS. Hep3B cells were grown in Eagle's Minimum Essential Medium (EMEM) supplemented with 10% FBS. Human umbilical vein endothelial cells (HUVECs) were cultured in a DMEM medium containing 20% FBS.

Plasmids, siRNA, and Cell Transfection

HHLA2 Expression: Human HHLA2-HA cDNA was synthesized by RuiBiotech Inc. (Guangzhou, China) and sequence-verified by Sanger sequencing. It was subsequently cloned into the pLenti-C-Myc-DDK-IRES-Puro lentiviral vector (OriGene, #PS100069) using Gibson Assembly (NEB, #M0530S). Primers used for HHLA2 amplification were: Forward: 5'-ATGAAGGCACAGACAGCACTGTCTT-3'; Reverse: 5'-TACTTTTCCTGAAAGAGGCACATTT-3'; **c-Met Expression:** The human *MET* coding sequence was PCR-amplified from cDNA derived from Huh7 cells (authenticated by STR profiling), sequence-verified, and cloned into the pLenti-CMV-GFP-Neo lentiviral vector (Addgene, #17447) using Gibson Assembly. Primers used for *MET* amplification were: Forward: 5'-ATGAAGGCCCCCGCTGTGCTTGCA CCTGGC-3'; Reverse: 5'-TGATGTCTCCC AGAAGGAGGCTGGTCGTGT-3'; **Kinase-Dead c-Met:** A kinase-dead (KD) c-Met mutant ($Y_{1234,1235}$F) was generated by site-directed mutagenesis using the QuikChange Site-Directed Mutagenesis Kit

(Agilent Technologies, #210518) with the wild-type (WT) pLenti-c-Met plasmid as template. Primers used for mutagenesis were: Forward: 5’-GTATGATAAAGAAT CTTCTAGTGTACACAACA-3’**;** Reverse: 5’-TGTTGTGTACACTAGAAGATTC TTTATCATAC-3’. **Plasmids for Hydrodynamic Injection (HDTVi):** The following plasmids, compatible with the Sleeping Beauty (SB) transposon system, were used for HDTVi: pT3-NRAS (Human NRAS; Addgene plasmid #20205); pT3-AKT (myr-AKT1; Addgene plasmid #31789); pCMV/SB11 (SB Transposase; Addgene plasmid #26552); pT3-c-Met (Human c-Met; Addgene plasmid #31784); pT3-HHLA2: The human HHLA2 coding sequence (synthesized as above) was first cloned into the pENTR™1A vector (Thermo Fisher Scientific, A10463) using In-Fusion HD Cloning (Takara Bio USA, #638918). Primers with homology arms for pENTR™1A were: Forward: 5′-TCAGTCGACTGGATCC ATGAAGGCACAGACAGCACTGTCTT-3′; Reverse: 5′-AAGCTGGGTCTAGATA TCCTATACTTTTCCTGAAAGAGGCACA-3′. HHLA2 was subsequently transferred from the pENTR-HHLA2 entry clone into the pT3 destination vector using the Gateway® LR Clonase™ II Enzyme mix (Thermo Fisher Scientific, 11791020) according to the manufacturer's protocol; pT3-c-Met-KD: The kinase-dead *MET* sequence was subcloned into the pT3 vector using similar Gateway cloning strategy.

siRNA

Negative control siRNA and pre-designed siRNAs targeting human HHLA2, MET, MMP2, and MMP9 were commercially synthesized by Tsingke Biotechnology Co., Ltd. (Guangzhou, China). The targeting SiRNA sequences are: MET: 5′-GGCCCAGCUU GCUAGACAA-3′; ICAM1:5′-GCCAACCAATGTGCTATTCAAAC-3′; MMP2: 5′-CCCCCAAAACGGACAAAGAGTTG-3′; MMP9: 5′-ACCACAACATCACCTATT GGATC-3′

Transfection

siRNA Transfection: Cells were transfected with siRNAs using Lipofectamine RNAiMAX Transfection Reagent (Thermo Fisher Scientific, 13778075) according to

the manufacturer's instructions. **Plasmid Transfection (for *in vitro* assays):** Plasmids were transfected into cells using Neofect™ DNA Transfection Reagent (NeosBioLab, TF201201, China) following the manufacturer's protocol. **Lentiviral Transduction:** For generating stable cell lines, lentiviral particles were produced using packaging plasmids in HEK293T cells and used to transduce target HCC cells, followed by selection with puromycin (Sigma-Aldrich, P8833) or neomycin (G418; Sigma-Aldrich, A1720) as appropriate.

Quantitative real-time polymerase chain reaction (qRT-PCR)

Total RNA was isolated from HCC cell lines using the FastPure Cell/Tissue Total RNA Isolation Kit (Vazyme, China) and reverse-transcribed into cDNA using the HiScript III RT SuperMix kit (Vazyme, China). qRT-PCR was performed on a CFX96 Touch Real-Time PCR Detection System (Bio-Rad, Hercules, CA, USA) using ChamQ Universal SYBR qPCR Master Mix (Vazyme, China). Specific primers were designed for target genes:

```
HHLA2: 5'-TACAAAGGCAGTGACCATTTGG-3',
       5'-AGGTGTAAATTCCTTCGTCCAGA-3'
MET:   5'-AGCAATGGGGAGTGTAAAGAGG-3';
       5'-CCCAGTCTTGTACTCAGCAAC-3,
MMP9:  5'-TGTACCGCTATGGTTACACTCG-3';
       5'-GGCAGGGACAGTTGCTTCT-3',
MMP2:  5'-TACAGGATCATTGGCTACACACC-3';
       5'-GGTCACATCGCTCCAGACT-3'
MMP7:  5'-GAGTGAGCTACAGTGGGAACA-3';
       5'-CTATGACGCGGGAGTTTAACAT-3'
CDH1:  5'-CGAGAGCTACACGTTCACGG-3';
       5'-GGGTGTCGAGGGAAAAATAGG-3',
CDH2:  5'-TCAGGCGTCTGTAGAGGCTT-3';
       5'-ATGCACATCCTTCGATAAGACTG-3',
VEGFA: 5'-AGGGCAGAATCATCACGAAGT-3';
       5'-AGGGTCTCGATTGGATGGCA-3',
GAPDH: 5'-GGAGCGAGATCCCTCCAAAAT-3';
       5'-GGCTGTTGTCATACTTCTCATGG-3'
```

GAPDH was used as an endogenous reference gene for normalization, and the data were analyzed using the 2^-ΔΔCT method.

Western blotting analysis

Cells were lysed in lysis buffers containing a complete protease inhibitor cocktail. Whole-cell lysates were clarified by centrifugation at 12,000 × g for 10 min. Protein concentrations in the supernatants were quantified using a Micro BCA™ Protein Assay Kit (Thermo Scientific, USA). Proteins were then separated by sodium dodecyl sulfate-polyacrylamide gel electrophoresis (SDS-PAGE) using 10% polyacrylamide gels and transferred onto polyvinylidene fluoride (PVDF) membranes (MilliporeSigma, Burlington, MA, USA). Membranes were blocked with 5% non-fat dry milk in Tris-buffered saline with Tween-20 (TBST) for 1-2 h at room temperature. Subsequently, membranes were incubated with primary antibodies diluted in TBST with 5% bovine serum albumin (BSA) overnight at 4 °C. After washing with TBST, membranes were incubated with the appropriate horseradish peroxidase (HRP)-conjugated secondary antibodies in TBST with 5% BSA for 30 min at room temperature. Protein bands were visualized using enhanced chemiluminescence (ECL) detection reagents (refer to the supplementary table for details on specific antibodies used).

Immunofluorescence (IF) staining

Cell lines: HCC cell lines were fixed with 4% paraformaldehyde, permeabilized with methanol, and subsequently blocked with 4% FBS in phosphate-buffered saline (PBS) for 30 min at room temperature. Cells were then incubated with primary antibodies diluted in PBS with 5% BSA overnight at 4 °C. After washing with PBS, cells were incubated with fluorescence-labeled secondary antibodies diluted in PBS with 5% BSA for 30 min at room temperature. Nuclei were counterstained with 4′,6-diamidino-2-phenylindole dihydrochloride (DAPI, Beyotime, China) for 5 min. Images were captured using a laser confocal microscope (Zeiss LSM880).

Immunohistochemical (IHC) staining

Formaldehyde-fixed, paraffin-embedded tissue sections were deparaffinized with xylene, rehydrated through a graded ethanol series, and treated with 0.3% hydrogen peroxide to inactivate endogenous peroxidase activity. Antigen retrieval was performed by steaming sections in EDTA buffer (pH 8.0) for 15 min. Sections were then incubated with the primary antibody diluted in PBS with 5% BSA overnight at 4 °C. After washing with PBS, sections were incubated with a horseradish peroxidase (HRP)-conjugated

secondary antibody (ZSGB-BIO, China) for 30 min at 37 °C. Immunoreactivity was visualized using DAB chromogenic substrate (Servicebio, China) under microscopic observation. Sections were then counterstained with hematoxylin.

HHLA2 and p-Met expression scoring: IHC staining intensity and the proportion of positive cells were used to semi-quantitatively evaluate HHLA2 or p-Met expression. The intensity of staining was graded on a scale of 0-3 (negative staining, light yellow, light brown, dark brown), and the proportion of positive cells was assessed on a scale of 1-4 (0-25%, 26-50%, 51-75%, 76-100%). The final staining score was calculated by multiplying the intensity score by the positive cell proportion score. Based on the median staining score, the samples were classified into high and low expression groups for HHLA2.Two experienced pathologists independently assessed the staining in a blinded manner, and mean percentage values were used for analysis.

CD34 (Microvessel Density - MVD) Scoring: To quantify angiogenesis, CD34 staining was assessed to determine MVD. Sections were first scanned at low magnification (e.g., 100x) to identify areas with the highest density of microvessels ("hotspots"). Within these hotspots, individual CD34-positive endothelial cells or cell clusters clearly separated from adjacent microvessels, tumor cells, or connective tissue elements were counted in 3-5 non-overlapping high-power fields (HPFs, e.g., 400x magnification). The average vessel counts per HPF or per mm^2 was calculated for each sample.

Co-immunoprecipitation (co-IP)

Cells were lysed in appropriate buffers containing a complete protease inhibitor cocktail. Whole-cell lysates were clarified by centrifugation at 12,000 × g for 10 min. For each co-immunoprecipitation reaction, 1 μg of specific antibody and 25 μL of protein A/G magnetic beads (Thermo Scientific, USA) were added to the lysates and incubated overnight at 4 °C with gentle rotation. The beads were then washed extensively with lysis buffer to remove unbound proteins. Finally, immune complexes were eluted from the beads by boiling in SDS-PAGE sample buffer for 5 min at 95 °C.

Mass spectrometry (MS) analysis

Following co-immunoprecipitation, the eluted proteins were separated by SDS-PAGE and visualized using silver staining (Beyotime, P0017). For MS analysis, protein bands of interest were excised from the gel and subjected to in-gel trypsin digestion. The

resulting peptides were analyzed by a timsTOF Pro mass spectrometer (Bruker) coupled to a NanoElute system (Bruker Daltonics) over a 60-minute gradient. The mass spectrometer was operated in positive ion mode. Raw MS data files were processed using Proteome Discoverer software (version 2.4.0.305; [RRID:SCR_014477]) and the built-in Sequest HT search engine (20) against the UniProt FASTA protein database ([RRID:SCR_002380]). A maximum of two missed cleavages per peptide was allowed during the database search. Peptide identifications were filtered at a false discovery rate (FDR) of 1%. All other search parameters were set to default.

Plasmids, siRNA and cell transfection

Negative control siRNA and siRNAs targeting HHLA2, c-Met, MMP2, and MMP9 were commercially synthesized by Tsingke Biotechnology Company (Guangzhou, China). siRNA transfections were performed using Lipofectamine RNAiMAX transfection reagent (Invitrogen, USA) according to the manufacturer's protocol. All plasmids were transfected into cells using Neofect transfection reagent (NeosBioLab, China) according to the manufacturer's instructions.

Cell viability assay

Cell viability was assessed using the Cell Counting Kit-8 (CCK-8; Beyotime, China) according to the manufacturer's instructions. Briefly, HCC cells were seeded in 96-well plates at a density of 1.0×10^3 cells per well in quintuplicate. After incubation for the indicated time points, 10 μL of the CCK-8 solution was added to each well, and the cells were incubated for an additional 1-4 hours following the manufacturer's recommendations. The absorbance at 450 nm was then measured using a Varioskan LUX multimode microplate reader (Thermo Scientific, USA). Higher absorbance values corresponded to greater cell viability.

Colony formation assays

Two-dimensional (2D) colony formation assay: HCC cells were seeded in triplicate at a density of 1.5×10^3 cells per well in six-well plates. After two weeks of incubation, cells were fixed with 4% paraformaldehyde for 30 min and stained with 0.5% crystal violet for 1 h. Colonies were visualized and counted under a microscope.

Soft agar colony formation assay: Anchorage-independent growth was assessed using a soft agar colony formation assay. The base layer consisted of 0.6% agar in a complete medium, solidified at room temperature. The top layer contained 0.3% agar in a

complete medium supplemented with 5.0 × 10^3 HepG2 cells per well. Cells were cultured for two weeks, and colonies were observed under a microscope.

Cell migration assay

As previously described, a silicone elastomer mask assay was used to quantify HCC cell migration[47]. Briefly, sterile silicone elastomer masks were placed in the center of each well in six-well plates to create a cell-free area. HCC cells (0.8 × 10^6 per well) were then seeded into the wells. After 24 hours of incubation, the masks were removed, and the medium was replaced with a serum-free medium to stimulate cell migration. Cell migration into the formerly cell-free area was monitored and photographed at 0, 24, and 48 hours using an inverted microscope. ImageJ software was used to quantify the area covered by migrated cells at each time point.

Transwell invasion assay

The invasive potential of HCC cells was assessed using a Transwell invasion assay with Matrigel chambers (Corning, USA). Briefly, HCC cells were seeded in triplicate at a density of 5.0 × 10^4 to 1.0 × 10^5 cells per well in the upper compartment of the Matrigel invasion chambers. The lower compartment contained 800 μL of DMEM supplemented with 10% FBS and 1 μM PHA665752 as a chemoattractant. After incubation for 24 or 48 hours, cells that invaded through the Matrigel to the lower surface of the membrane were fixed with 4% paraformaldehyde, stained with 0.5% crystal violet, and visualized under a microscope.

Preparation of conditioned medium:

HCC cells were seeded at a density of 1.0 × 10^6 cells per well in six-well plates. To investigate the effects of PHA665752 and si-Met on HCC cell-derived factors, cells were cultured overnight in serum-free medium with or without 1 μM PHA665752 treatment or transfected with either control siRNA or si-Met targeting c-Met. After 24 hours, the conditioned medium was collected and centrifuged to remove cellular debris. The resulting supernatant was then used for subsequent experiments, including ELISA and HUVEC tube formation assays.

HUVEC tube formation assay

The angiogenic potential of the conditioned medium was assessed using a HUVEC tube formation assay. Briefly, 96-well plates were pre-cooled and coated with 50 μL/well of Matrigel (Corning, USA). The Matrigel was allowed to solidify at 37 °C for 30 min.

HUVECs were harvested using trypsin and resuspended in a serum-free medium. Aliquots of 100 μL of cell suspension (containing 2.0×10^4 cells/well) were then plated onto the solidified Matrigel. The plates were incubated for 6 hours at 37 °C in a humidified incubator containing 5% CO_2. Five images were captured per well using an inverted microscope. ImageJ software quantified the number of tube-like structures formed by the HUVECs. Experiments were performed in triplicate and independently repeated three times.

RNA sequencing analysis

Total RNA was isolated from HepG2 cells treated with control medium (n = 3) or medium overexpressing HHLA2 (OE-HHLA2; n = 3) using established protocols. RNA sequencing libraries were then constructed and sequenced on the Illumina NovaSeq6000 (Biomarker Technologies sequencing platform) using paired-end 150 bp reads (PE150). Briefly, polyadenylated mRNA was enriched from total RNA using oligo(dT) magnetic beads. The isolated mRNA was fragmented, reverse transcribed into cDNA, and purified. The fragmented cDNA was end-repaired, A-tailed, and ligated with sequencing adapters. Following library size selection and quality control, libraries were sequenced using the Illumina NovaSeq6000 platform. Sequencing data were analyzed using a standard pipeline, including quality control, read alignment to the human genome, gene quantification, and differential expression analysis. Genes were considered differentially expressed if they exhibited a $|\log2(\text{fold change})|$ exceeding 0.4 and a p-value less than 0.05.

VEGFA Enzyme-linked immunosorbent assay (ELISA)

Soluble VEGFA protein levels in cell culture supernatants were quantified using a commercially available Human VEGF ELISA Kit (Beyotime, China) according to the manufacturer's protocol. Briefly, samples and standards were incubated in ELISA plates pre-coated with capture antibodies specific for VEGFA. After washing steps to remove unbound substances, a horseradish peroxidase (HRP)-conjugated detection antibody was added. The amount of bound HRP-conjugated antibody was then measured using a chromogenic substrate with absorbance measured at 450 nm. The concentration of VEGFA in the samples was extrapolated from a standard curve generated using known concentrations of recombinant human VEGF.

Gelatin Zymography

To assess MMP9 enzymatic activity, gelatin zymography was performed on conditioned media (CM). HepG2-Vec and HepG2-HHLA2 cells were cultured to confluence in 60 mm plates and then incubated in serum-free medium for 24-48 hours. The collected CM was centrifuged to remove cell debris and concentrated approximately 20-fold using Amicon® Ultra centrifugal filter units with a 50 kDa molecular weight cutoff (MilliporeSigma). Equal volume of CMs were mixed with non-reducing sample buffer and resolved on 8% SDS-PAGE gels containing 1 mg/mL gelatin (Sigma-Aldrich). After electrophoresis, gels were washed twice for 30 min in renaturation buffer (2.5% Triton X-100) and incubated overnight at 37°C in developing buffer (50 mM Tris-HCl pH 7.5, 200 mM NaCl, 5 mM $CaCl_2$, 0.02% Brij-35). Gels were stained with 0.5% Coomassie Brilliant Blue R-250 (Bio-Rad) and destained. Gelatinolytic activity, primarily corresponding to MMP9 (~92 kDa), appeared as clear bands on a blue background and was quantified using densitometry (ImageJ).

Split luciferase complementation assay

To investigate the direct physical interaction between HHLA2 and c-Met, and to identify the interacting domains, we employed a split-luciferase complementation assay. we utilized the NanoLuc Binary Technology (NanoBiT) system from Promega. We created stable HEK293 cell lines expressing either LgBiT-tagged HHLA2 or LgBiT-tagged c-Met. The LgBiT fragment and a flexible linker (LgBiT-GSSGGGGSGGGGSSG-) were inserted downstream of the signal peptide (sp) sequences of full-length HHLA2 (between amino acid 23 and 24) or c-Met (between amino acid 25 and 26), ensuring surface expression of the tagged proteins. These constructs (sp-LgBiT-Linker-HHLA2 and sp-LgBiT-Linker-c-MET) were cloned into the lentiviral expression vector plenti-IRES-Puro (Origene, PS100069) for stable cell line generation. Lentiviral particles were produced according to manufacturer instructions and used to transduce HEK293 cells. Stable cell lines were selected using puromycin (1 μg/mL).

For the assay, cells expressing the LgBiT-tagged proteins were seeded in 96-well plates. Recombinant proteins containing the extracellular domains (ExD) of either HHLA2 or c-Met, fused to the SmBiT fragment and a 6xHis tag (HHLA2ExD-SmBiT-6His or c-METExD-SmBiT-6His), were added to the wells at various concentrations. These recombinant proteins were produced in Sf9 cells using the BacMam expression system and purified via Ni-NTA chromatography (details provided in the 'Recombinant protein expression and purification' section).

After a 1-hour incubation period to allow for protein-protein interaction and subsequent LgBiT/SmBiT complementation, Nano-Glo Live Cell Substrate (Promega) was added according to the manufacturer's instructions. This substrate is specifically designed for use with NanoLuc luciferase. Luminescence, which is directly proportional to the amount of reconstituted luciferase and thus to the extent of HHLA2-c-Met interaction, was measured using a Varioskan LUX multimode microplate reader (Thermo Scientific) after a 15-minute incubation period. All assays were performed in triplicate, and data are presented as relative luminescence units (RLU)

Protein in vitro binding assay

The in vitro interaction between HHLA2 and c-met was examined using two methods. In the first method, 20 μg purified HHLA2ExD-SmBit-6His and c-MET-ExD-SmBit-6His recombinant proteins were incubated with anti-HHLA2-agarose beads at 4°C overnight. After washing three times with pre-cold PBS buffer to remove unbound proteins, the presence of c-MET was detected using an anti-c-MET antibody. In the second method, 1 μg/well of purified c-MET-ExD-SmBit-6His protein was cross-linked overnight to the bottom of a 96-well plate. Then, 1 μg to 20 μg of HHLA2ExD-SmBit-6His protein was added to each well and incubated for one hour. After three washes with PBS to remove unbound HHLA2, the amount of HHLA2 bound to c-met in the wells was quantified using an ELISA kit (CwBio, China, CW0050).

Animal models and in vivo experiments

All animal experiments were performed according to protocols approved by the Animal Care and Use Committee of Nanjing Drum Tower Hospital and adhered to the Guide for the Care and Use of Laboratory Animals. Sample sizes for animal studies (n=5-7

per group for xenograft models; n=6 per group for metastasis models; n=11 per group for the HDTVi model, as indicated in figure legends) were chosen based on previous experience with these specific HCC models in our laboratory and similar published studies, aiming to provide sufficient statistical power to detect biologically meaningful differences while adhering to the principles of the 3Rs (Replacement, Reduction, Refinement). Following tumor cell inoculation or hydrodynamic injection, mice were randomly assigned to control or treatment groups using a random number generator to minimize allocation bias. Furthermore, investigators responsible for administering treatments, monitoring animal health, measuring tumor growth (where applicable), performing imaging, conducting endpoint tissue processing, and quantifying histological or molecular data were blinded to the experimental group assignments. Blinding was maintained throughout the experimental and data analysis process until final statistical comparisons were performed.

Mice

Five-week-old male BALB/c nude mice and seven-week-old male C57BL/6 mice were purchased from Guangdong Medical Laboratory Animal Center and housed in specific pathogen-free facilities.

In vivo models

Intrahepatic xenograft models**:** Stable HCC cell lines (HepG2-vec and HepG2-HHLA2) were established and inoculated into the left lateral lobe of the liver (2 million cells per mouse) of nude mice. Tumor growth was monitored by bioluminescence imaging (IVIS Lumina XRMS, PerkinElmer) four weeks after inoculation. Prior to imaging, mice were injected intraperitoneally with D-luciferin (Gold Biotechnology, catalog number: LUCNA-1G), the substrate for firefly luciferase, at a dose of 150 mg/kg body weight, dissolved in sterile PBS. Mice were anesthetized with isoflurane, and imaging was performed 10-15 minutes after luciferin injection, allowing for sufficient substrate distribution. Bioluminescence signal was quantified using Living Image software (PerkinElmer) by drawing regions of interest (ROIs) around the liver, and total flux (photons/second) was measured. Mice were then sacrificed. Mice were then sacrificed, and livers were harvested, weighed, imaged, and processed for further analysis. Specifically, a portion of the tumor tissue and adjacent liver lobe were fixed in 4% paraformaldehyde for hematoxylin and eosin (H&E) and immunohistochemistry (IHC)

staining, while the remaining tissues were snap-frozen in liquid nitrogen for subsequent analysis.

Hydrodynamic co-expression models: To model NRAS/AKT-driven HCC *in vivo*, plasmids encoding various components were delivered via hydrodynamic tail vein injection (HDTVi) into eight-week-old C57BL/6 mice. This method allows for efficient gene delivery primarily to hepatocytes. The myr-AKT1 + N-RasV12-driven HDTVi model, known to rapidly induce HCC development recapitulating key features of human disease [cite relevant supporting paper(s)], was used to assess the impact of HHLA2.

Specifically, combinations included:

- Control Group: pCDNA3.1-Myc and Plasmids encoding myr-AKT1 and N-RasV12, delivered together with the Sleeping Beauty (SB) transposase plasmid (pT3/pCMV-SB) to ensure genomic integration.
- HHLA2 Overexpression Group: Plasmids encoding myr-AKT1, N-RasV12, and myc-tagged HHLA2, delivered together with the SB transposase plasmid.
- c-Met Dependency Groups (Figure 4G/H): Plasmids encoding myr-AKT1, N-RasV12, myc-HHLA2 (or control vector), SB transposase, and either wild-type human c-Met (pT3-c-Met) or a kinase-dead human c-Met mutant (pT3-c-Met-KD; Y1234F/Y1235F).
- c-Met Inhibition Groups (Figure 5G-J): Similar to the HHLA2 overexpression group, with subsequent pharmacological inhibition using PHA665752 (details in 'C-Met inhibitor treatment' section) or co-injection of the kinase-dead c-Met mutant (MET-KD) plasmid as specified.

As HHLA2's oncogenic effects in this context appear dependent on activating c-Met and cooperating with established drivers like AKT/Ras, a group receiving only HHLA2 (which was not expected to induce rapid tumorigenesis alone) was not included in this study design focused on HHLA2's interaction with established HCC drivers.

Monitoring and humane endpoints: All mice were monitored for abdominal girth and signs of morbidity or discomfort throughout the experiment. Humane endpoints included reaching a maximum tumor size not exceeding 2 cm (though often difficult to measure discretely in this model, see below) or a significant loss of body weight (exceeding 20% of initial weight). Mice were sacrificed when endpoints were met or at designated time points for analysis. Due to the nature of the HDTVi model, tumors often develop diffusely throughout the liver, making the counting of discrete nodules and measurement of individual tumor diameters impractical. Therefore, the primary metric used to assess overall tumor burden in this model was the liver-to-body weight ratio, calculated after sacrifice. Sample sizes were determined based on previous experience and pilot studies indicating group sizes of n=6-11 would provide sufficient power. Animals were randomly assigned to groups, and investigators were blinded during experiments and outcome assessment.

C-Met inhibitor treatment: Mice were treated with c-Met inhibitor PHA665752 (20 mg/kg) or vehicle (physiological saline) intraperitoneally every other day.

Tissue dissociation and organoid culture

Fresh liver tumor tissue was obtained from patients undergoing hepatectomy for HCC. Blood, fat, necrotic tissue, and connective tissue were meticulously removed to enrich tumor cells. The remaining tissue was then dissected into small pieces. The tissue fragments were washed in 5 mL of 5 mM PBS/EDTA for 15 minutes at room temperature to remove residual blood. Subsequently, the tissue was incubated in 5 mL of 1 mM PBS/EDTA containing 2x TrypLE for one hour at 37 °C to promote enzymatic dissociation. The dissociated cell clusters were liberated from the remaining tissue fragments by applying gentle mechanical force with a pipette. The resulting cell suspension was centrifuged at 350 g to pellet the cells. The cell pellet was then resuspended in a mixture of 50% Matrigel and organoid culture medium at a density of $2X10^5$/mL. Aliquots of 100 μL of the cell suspension were plated onto a 24-well plate. The plates were incubated for 30 minutes at 37 °C and 5% CO_2 for Matrigel solidification. Following solidification, 1 mL of fresh organoid culture medium was added to each well. The culture medium was replaced every 72 hours.

Organoid drug response assay

The c-Met inhibitor PHA665752 (15 μM, HY-15735, MCE) or dimethyl sulfoxide (DMSO, vehicle control) was added to the PDOs. After 48 hours, cell viability and apoptosis were assessed using the Calcein-AM/PI Live/Dead Cell Double Staining Kit (G1707, Servicebio) according to the manufacturer's instructions. Stained organoids were imaged with a high-content imaging analysis system (AMOview-100, Amoolo Biotech), detecting Calcein at 490/515 nm and PI at 535/617 nm. Fluorescence ratios indicating cell viability and apoptosis were normalized to vehicle-treated controls.Organoid culture supernatant was collected 48 hours post-treatment, and cell damage was evaluated using the CyQUANT™ LDH Cytotoxicity Assay Kit (C20300, Thermo Fisher) as per the manufacturer's protocol.

Flow cytometry analysis of immune cell infiltrates

Liver tissues were mechanically dissociated using a mortar and pestle to isolate infiltrating immune cells. Single-cell suspensions were washed with PBS containing 1% FBS and red blood cells were lysed using an appropriate lysis buffer. Cells were then stained with fluorochrome-conjugated antibodies specific for various immune cell surface markers in the same buffer. The following antibodies were used:

- Anti-human CD45 (to identify human leukocytes)
- Anti-mouse CD19 (B cells)
- Anti-mouse CD11c+ and MHCII+ (dendritic cells)
- Anti-mouse CD11b+ (monocytes and Kupffer cells)
- Anti-mouse CD11b+ and F4/80+ (Kupffer cells)
- Anti-mouse CD11b+ and Ly6G+ (neutrophils)
- Anti-mouse NK1.1 (NK cells)
- Anti-mouse CD107a+ and NK1.1+ (activated NK cells)
- Anti-mouse CD8+ (CD8+ T cells)
- Anti-mouse CD69+ and CD8+ (activated CD8+ T cells)
- Anti-mouse CD4+ (CD4+ T cells)
- Anti-mouse CD69+ and CD4+ (activated CD4+ T cells)

Flow cytometry was analyzed using a BD FACSAria™ III Cell Sorter (BD Bioscience, USA). Data were analyzed using FlowJo software v.10.

Statistical analysis

Statistical analyses were performed using GraphPad Prism version 10 (La Jolla, CA, USA). For comparisons between two groups, unpaired or paired Student's t-tests were used as appropriate based on experimental design. For comparisons between multiple groups, one-way or two-way analysis of variance (ANOVA) followed by post-hoc tests (e.g., Tukey's multiple comparisons test) were used. Normality of data distribution was assessed using appropriate tests (e.g., the Shapiro-Wilk test). Data were presented as mean ± standard deviation (SD) or median with interquartile range (IQR) depending on the distribution of the data. P-values less than 0.05 ($p < 0.05$) were considered statistically significant.

Supplemental Tables

Supplemental Table 1. Correlation between HHLA2 Expression and Clinicopathological Factors in 176 HCC Patients from Guangdong, China

Characteristics	No. of patients	HHLA2 expression (%)		*p*-value
		Low	High	
Gender				
Female	21	7 (33.3%)	14 (66.7%)	
Male	155	76 (49.0%)	79 (51.0%)	0.176
Age (years)				
≤ 50	93	38 (40.9%)	55 (59.1%)	
> 50	83	45 (54.2%)	38 (45.8%)	0.076
AFP (ng/ml)				
≤ 400	101	47 (46.5%)	54 (53.5%)	
> 400	75	36 (48.0%)	39 (52.0%)	0.847
HBsAg				
Negative	11	6 (54.5%)	5 (45.5%)	
Positive	165	77 (46.7%)	88 (53.3%)	0.612
Liver cirrhosis				
No	37	21 (56.8%)	16 (43.2%)	
Yes	139	62 (44.6%)	77 (55.4%)	0.188
Tumor size (cm)				
≤ 5	102	62 (60.8%)	40 (39.2%)	
> 5	74	21 (28.4%)	53 (71.6%)	**< 0.001**
Tumor number *				
Single	164	77 (47.0%)	87 (53.0%)	
Multiple	12	6 (50.0%)	6 (50.0%)	0.838
Satellite nodule				
No	155	75 (48.4%)	80 (51.6%)	
Yes	21	8 (38.1%)	13 (61.9%)	0.375
Tumor capsule				
No/incomplete	105	45 (42.9%)	60 (57.1%)	
Complete	71	38 (53.5%)	33 (46.5%)	0.164
Tumor differentiation				
I-II	125	56 (44.8%)	69 (55.2%)	
III-IV	51	27 (52.9%)	24 (47.1%)	0.326
Vascular invasion				
No	157	79 (50.3%)	78 (49.7%)	
Yes	19	4 (21.1%)	15 (78.9%)	**0.016**
BCLC stage				
0	16	10 (62.5%)	6 (37.5%)	
A	80	50 (62.5%)	30 (37.5%)	
B	62	17 (27.4%)	45 (72.6%)	
C	18	6 (33.3%)	12 (66.7%)	**< 0.001**
Recurrence or metastasis				
Negative	80	54 (67.5%)	26 (32.5%)	
Positive	96	29 (30.2%)	67 (69.8%)	**< 0.001**

* Single or multiple HCC: either one tumor or two and above tumors within the liver

Supplemental Table 2. Univariate and Multivariate Analyses of Postoperative Prognosis in 176 HCC Patients

	OS				TTR			
	Univariate	Multivariate			Univariate	Multivariate		
Variables*	*p*-value	*p*-value	HR	95% CI	*p*-value	*P*-value	HR	95% CI
Gender (Female vs. Male)	NS	NS			NS	NS		
Age, years (≤ 50 vs. > 50)	NS	NS			NS	NS		
AFP (ng/mL) (≤ 400 vs. > 400)	< 0.001	< 0.001	2.539	1.623-3.973	< 0.001	< 0.001	2.756	1.794-4.235
HBsAg (Negative vs. Positive)	NS	NS			NS	NS		
Liver cirrhosis (No vs. Yes)	0.003	0.009	2.586	1.272-5.257	0.003	0.021	2.142	1.124-4.083
Tumor size (cm) (≤ 5 vs. > 5)	< 0.001	NS			< 0.001	NS		
Tumor number (Single vs. Multiple)	NS	NS			NS	NS		
Satellite nodule (No vs. Yes)	NS	NS			0.029	NS		
Tumor capsule (No/ incomplete vs. Complete)	NS	NS			NS	NS		
Tumor differentiation (I-II vs. III-IV)	NS	NS			NS	NS		
Vascular invasion (No vs. Yes)	< 0.001	< 0.001	5.169	2.792-9.569	< 0.001	< 0.001	4.673	2.630-8.304
HHLA2 (Low *versus* High)	< 0.001	0.004	2.093	1.261-3.475	< 0.001	< 0.001	2.758	1.719-4.426

Supplemental Table 3 Number and Proportion of Intrahepatic Metastases in 4 Groups of Orthotopic Liver xenograft Model.

	Intrahepatic metastasis
HepG2-vec	0/5
HepG2-HHLA2	2/5
HepG2-vec-PHA665752	0/5
HepG2-HHLA2-PHA665752	0/5

Supplemental Table 4. Correlation between HHLA2 Expression and p-MET levels of a cohort of 71 HCC patients from Shanghai, China

		NO. expression of p-MET(%)			
		-/+	++/+++	Total	P-value
HHLA2	-/+	39(54.93%)	4(5.63%)	43(60.56%)	
	++/+++	10(14.08%)	18(25.36%)	28(39.44%)	
	Total	49(69.01%)	22(30.99%)	71(100%)	P<0.0001

Supplemental Figure Legends

Supplemental Figure 1. HHLA2 expression correlates with survival in patients with HCC. (A) Analysis of HHLA2 mRNA expression. *Top panels:* Comparison of HHLA2 mRNA levels in HCC versus Normal Liver tissues using data from Gene Expression Omnibus (GEO) datasets GSE64485 (n=50 HCC vs 5 Normal; *left*) and GSE190174 (n=5 HCC vs 5 Normal; *right*). P values were determined by Student's t-test. *Bottom panel:* Pan-cancer analysis displaying HHLA2 mRNA expression levels (log2 TPM) across various tumor types from The Cancer Genome Atlas (TCGA) and GTEx databases compared to corresponding available normal tissues (Data source: TIMER2.0). **(B)** HHLA2 mRNA expression (TPM) in HCC samples from the TCGA-LIHC project, stratified by pathological tumor grade (n = 371 individuals with grade information). *P* values determined by unpaired two-tailed t test **(C)** Kaplan-Meier analysis of overall survival (OS) in HCC patients from the TCGA-LIHC project (n = 371 individuals with survival data) stratified by high versus low HHLA2 mRNA expression (median cutoff). *P* value determined by log-rank test. **(D)** Forest plot showing multivariate Cox regression analysis of OS in the TCGA-LIHC cohort (analysis includes n = 357 individuals with complete data for all variables in the model). Hazard ratios (HR) and 95% confidence intervals (CI) are shown. *P* values determined by Wald's test. Patient data in (C-E) are from TCGA-LIHC project, n = 357 individuals with grade information. **(E)** HHLA2 protein levels assessed by immunohistochemical staining in HCC and paired normal tissues from the Guangdong cohort (n = 176 cases). P values were determined by paired two-tailed t test. **(F)** Kaplan-Meier survival curves for overall survival and recurrence-free Survival in the Guangdong HCC cohort (n = 176 individuals), stratified by tumor size (≤5 cm or >5 cm), tumor differentiation grade, and Barcelona Clinic Liver Cancer (BCLC) stage. P values were determined by log-rank test. * $P < 0.05$, ** $P < 0.01$, *** $P < 0.001$, **** $P < 0.0001$.

Supplemental Figure 2. HHLA2 promotes HCC cell growth in vitro. (A, B) HHLA2 mRNA (A) and protein (B) expression in HCC cell lines. (C-E) Western blot validation of HHLA2

overexpression in HepG2 (C) and Hep3B (D)cells and HHLA2 knockdown in Huh7 (E) cells. All Western blots shown are representative of three independent experiments; relative quantification values are indicated below the bands, and full quantitative data/statistical analysis are provided in the supplementary Excel spreadsheet. (F, G) Cell proliferation assessed by CCK-8 assay in Hep3B (F)and Huh7 (G) cells with HHLA2 overexpression or knockdown. P values were determined by one-way ANOVA. (H-J) Two-dimensional colony formation assays in HepG2 (H), Hep3B (I), and Huh7 (J) cells with HHLA2 overexpression or knockdown (2 weeks). (K) Soft agar colony formation assay in HepG2-Vec and HepG2-HHLA2 cells. P values were determined by two-tailed Student's t test. * $P < 0.05$, ** $P < 0.01$, *** $P < 0.001$, **** $P < 0.0001$. Scale bars, 100 μm

Supplemental Figure 3. HHLA2 promotes aggressive phenotypes in HCC. (A) Cell proliferation assessed by CCK-8 assay in the indicated Huh7 cells. (B, C) Cell migration assays in Hep3B (B) and Huh7 (C) cells with HHLA2 overexpression or knockdown. (D, E) Transwell invasion assays in Hep3B (D) and Huh7 (E) cells with HHLA2 overexpression or knockdown. (F, G) HUVEC tube formation assays using conditioned media from Hep3B (F) or Huh7 (G) cells with HHLA2 overexpression or knockdown. (H) Representative images and quantification of liver tumor sizes in orthotopic xenografts derived from Huh7 cells expressing either a nontargeting shRNA (Huh7-shNC) or an shRNA targeting HHLA2 (Huh7-HHLA2KD). $n = 6$ mice per group. P values were determined by one-way ANOVA (A) or two-tailed Student's t test (B–H). * $P < 0.05$, ** $P < 0.01$, *** $P < 0.001$, **** $P < 0.0001$. Scale bars, 100 μm

Supplemental Figure 4. HHLA2 expression modulates specific proteins and pathways. (A) Heatmap analysis of differentially expressed proteins and phosphoproteins in HCC samples with high or low HHLA2 expression from TCGA and The Cancer Proteome Atlas (TCPA) databases. The heatmap displays proteins and phosphoproteins with a fold change greater than 1.2 between high and low HHLA2–expressing HCC samples. (B) Kyoto Encyclopedia of Genes and Genomes (KEGG) pathway enrichment analysis of differentially expressed proteins identified in (A). Only pathways with $P < 0.05$ are shown. (C) Volcano plot of differentially expressed mRNAs in HepG2 cells following HHLA2 overexpression. Genes with $|\log2$ (fold change) $| > 0.4$ and $P < 0.05$ are highlighted ($n = 3$ samples per group). (D) Gene set enrichment analysis (GSEA) of cell proliferation processes. GSEA was performed on the differentially expressed genes identified in (C). NES, normalized enrichment score.

Supplemental Figure 5. HHLA2 interacts directly with c-Met. (A) Coimmunoprecipitation of overexpressed HHLA2-HA and endogenous c-MET in Hep3B cells. (B) Coimmunoprecipitation of endogenous c-MET and HHLA2 in HepG2 cells. The antibodies used for immunoprecipitation (IP) and Western blot are indicated. (C) Split luciferase complementation assay to define the interaction domains between HHLA2 and c-Met. Luminescence was measured following the addition of indicated amounts of recombinant HHLA2-ExD SmBit-6His to 293-LgBiT-MET cells. (D) Coimmunoprecipitation of HHLA2-HA with endogenous c-Met in HepG2-Vec or HepG2-HHLA2 cells treated with 1 μg/ml tunicamycin (N-linked glycosylation inhibitor) or DMSO (control) for 24 hours. (E, F) In vitro interaction between N-linked glycosylated or unglycosylated HHLA2 and c MET assessed by split luciferase complementation assay. Luminescence was measured following the addition of indicated concentrations of glycosylated or unglycosylated HHLA2-ExD-SmBit-6His to 293-LgBiT-HHLA2 or 293-LgBiT-MET cells, respectively (E). P values were determined by two-tailed Student's t test. **** $P < 0.0001$. Ponceau S staining of input HHLA2-ExD-SmBit-6His proteins is shown in F. (G-I) Western blot analysis of c-MET signaling pathway proteins in Hep3B cells with or without HHLA2 overexpression (G) and Huh7 cells with or without HHLA2 knockdown and rescue under normal (H) or starvation (I) conditions. All Western blots shown are representative of three independent experiments; relative quantification values are

indicated below the bands, and full quantitative data/statistical analysis are provided in the supplementary Excel spreadsheet.

Supplemental Figure 6. MMP family and VEGFA-VEGFR2 signaling contribute to HHLA2-driven invasion and tumor angiogenesis. (A–D) qRT-PCR analysis of HHLA2, E-cadherin, and N-cadherin mRNA expression (A); Western blot analysis of the indicated protein levels (B); representative bright-field images showing morphology (C); and qRT-PCR analysis of MMP2 mRNA expression (D) in HepG2-Vec and HepG2-HHLA2 cells. (E–G) Representative images (E) and quantification (F) of invading HepG2-Vec and HepG2-HHLA2 cells with or without MMP2 knockdown; MMP2 knockdown efficacy was assessed by qRT-PCR (G). (H, I) qRT-PCR analysis of MMP9 mRNA expression in Hep3B-Vec and Hep3B-HHLA2 cells (H) and Huh7 cells with or without HHLA2 knockdown and rescue (I). **(J)** Gelatin zymography analysis of conditioned media from HepG2-Vec and HepG2-HHLA2 cells, demonstrating increased MMP9 enzymatic activity upon HHLA2 overexpression. **(K)** Western blot validation of MMP9 knockdown in HepG2-Vec and HepG2 HHLA2 cells. **(L)** Transwell invasion assays of Huh7 and Huh7-HHLA2KD cells with or without MMP9 knockdown. **(M, N)** qRT-PCR analysis of VEGFA mRNA expression in Hep3B cells with or without HHLA2 overexpression (M) and Huh7 cells with or without HHLA2 knockdown and rescue (N). **(O, P)** ELISA analysis of VEGFA secretion in Hep3B cells with or without HHLA2 overexpression (O) and Huh7 cells with or without HHLA2 knockdown and rescue (P). **(Q)** HUVEC tube formation assays using conditioned media from Hep3B Vec or Hep3B-HHLA2 cells, with or without treatment with 50 ng/ml apatinib (VEGF receptor inhibitor). Western blots and Gelatin zymography shown are representative of three independent experiments; relative quantification values are indicated below the bands, and full quantitative data/statistical analysis are provided in the supplementary Excel spreadsheet. P values were determined by two-tailed Student's t test. * $P < 0.05$, ** $P < 0.01$, *** $P < 0.001$, **** $P < 0.0001$. Scale bars, 100 μm.

Supplemental Figure 7. c-Met activation is required for HHLA2-mediated MMP9 and VEGFA expression and secretion. (A–C) qRT-PCR analysis of MMP9 and VEGFA mRNA expression in (A) HepG2-Vec and HepG2-HHLA2 cells, (B) Hep3B-Vec and Hep3B-HHLA2 cells, and (C) Huh7-Vec and Huh7-HHLA2KD cells. All cell lines were treated with or without 100 nM PHA665752 (c-Met inhibitor). (D, E) ELISA of VEGFA secretion in HepG2-Vec and HepG2-HHLA2 cells with or without 100 nM PHA665752 treatment (D) and with or without c MET knockdown (E). (F–H) Western blot analysis of ERK activation, VEGFA, and MMP9 expression in (F) HepG2-Vec and HepG2-HHLA2 cells, (G) Hep3B-Vec and Hep3B-HHLA2 cells, and (H) Huh7-Vec and Huh7 HHLA2KD cells. All cell lines were treated with or without 100 nM PHA665752. (I, J) Western blot analysis of ERK activation, VEGFA, and MMP9 expression in (I) HepG2-Vec and HepG2-HHLA2 cells and (J) Huh7-Vec and Huh7-HHLA2KD cells. All cell lines were treated with or without c-MET knockdown. All Western blots shown are representative of three independent experiments; relative quantification values are indicated below the bands, and full quantitative data/statistical analysis are provided in the supplementary Excel spreadsheet. P values were determined by two-tailed Student’s t test. ns, not significant; * $P < 0.05$, ** $P < 0.01$, *** $P < 0.001$, **** $P < 0.0001$. Scale bars, 100 μm

Supplemental Figure 8. c-Met inhibition by PHA665752 abrogates HHLA2-mediated HCC progression in vitro. (A, B) Cell proliferation assessed by CCK-8 assay in HepB3-Vec and HepB3-HHLA2 cells (A) or Huh7-Vec and Huh7-HHLA2KD cells (B). (C, D) Two-dimensional colony formation assay in HepB3-Vec and HepB3-HHLA2 cells (C) or Huh7-Vec and Huh7-HHLA2KD cells (D). (E, F) Cell migration assay in HepB3-Vec and HepB3 HHLA2 cells (E) or Huh7-Vec and Huh7-HHLA2KD cells (F). (G) Transwell invasion assay of Huh7-Vec and Huh7-HHLA2KD cells. (H, I) HUVEC tube formation assay induced by conditioned media from HepB3-Vec and HepB3-HHLA2 cells (H) or Huh7-Vec and Huh7-HHLA2KD cells (I). All cells were treated with 100 nM PHA665752 or DMSO as indicated. P values were determined by two-tailed Student’s t test. ns, not significant; * $P < 0.05$, ** $P < 0.01$, *** $P < 0.001$, **** $P < 0.0001$. Scale bars, 100 μm.

Supplemental Figure 9. Clinically relevant c-Met inhibitor cabozantinib abrogates HHLA2-mediated aggressive phenotypes *in vitro*. (A-E) Assessment of malignant phenotypes in HepG2-Vec and HepG2-HHLA2 cells, with or without 1 μM cabozantinib treatment. Phenotypes evaluated include (A) cell proliferation by CCK-8 assay, (B) two-dimensional colony formation, (C) cell migration assay using a wound-healing approach, (D) Transwell invasion assay, and (F) HUVEC tube formation using conditioned media from treated HepG2 cells. P values were determined by one-way ANOVA for (A), or two-tailed t test for (B-E). * P < 0.05, ** P < 0.01, *** P < 0.001, **** P < 0.0001. Scale bars, 100 μm.

Supplemental Figure 10. c-Met knockdown abrogates HHLA2-mediated HCC progression in vitro. (A C) Cell migration assays of HepG2-Vec and HepG2-HHLA2 cells (A), HepB3-Vec and HepB3-HHLA2 cells (B), or Huh7-Vec and Huh7-HHLA2KD cells (C) with or without c-MET knockdown. (D) Transwell invasion assay of HepG2-Vec and HepG2-HHLA2 cells with or without c-MET knockdown. (E, F) HUVEC tube formation assays induced by conditioned media from HepG2-Vec and HepG2-HHLA2 cells (E) or Huh7-Vec and Huh7-HHLA2KD cells (F) with or without c-MET knockdown. P values were determined by two-tailed Student's t test. ns, not significant; * P < 0.05, ** P < 0.01, **** P < 0.0001. Scale bars, 100 μm

Supplemental Figure 11. Infiltration of immune cell subsets in mouse tumor tissue. (A) Representative images of orthotopic liver tumors in C57BL/6 mice following HDTVi delivery of myc-HHLA2, a control vector, wild type (WT) c-MET, or kinase-dead (KD) c-MET. (A) Representative images of liver tumors in orthotopic xenograft models in nude mice injected with HepG2-HHLA2 cells, followed by intraperitoneal administration of PHA665752 (n = 5 mice per group). (B) Representative images of lung tissues from nude mice following tail vein injection of HepG2-Vec or HepG2-HHLA2 cells, with or without PHA665752 treatment every other day for 28 days. Lung tissues were assessed for HCC metastasis. (C-E) Flow cytometry analysis of immune cell subpopulations in C57BL/6 mouse livers following HDTVi delivery of Myc-HHLA2 or a control vector, along with N-RasV12/myr AKT1 and the Sleeping Beauty

transposon system. (C) Representative flow cytometry plots showcasing various immune cell populations (macrophages, neutrophils, CD4+ T cells, CD8+ T cells, and B cells) in the liver. (D) Quantification of the relative abundance of each immune cell subpopulation. (E) Changes in indicated immune cell activity in different treatment groups. Data are presented as mean ± SD (n = 5 mice per group). P values were determined by two-tailed Student's t test. ns, not significant; * $P < 0.05$, ** $P < 0.01$, **** $P < 0.0001$. Scale bars, 100 μm

Supplemental Figure 12. HHLA2 expression predicts efficacy of c-Met inhibitor therapy in HCC. (A–C) Correlation between c-Met/HHLA2 expression and c-Met inhibitor efficacy in HCC cell lines. Relationship between c-Met expression level (A), HHLA2 expression level (B), or HHLA2 expression levels with abnormal c Met expression. Scoring methods are detailed in the Supplemental Methods section. P value was determined by Pearson correlation analysis. $P < 0.0001$. (C) and the efficacy of the c-Met inhibitor PF2341066 in cancer cell lines from the Cancer Cell Line Encyclopedia (CCLE) database. (D, E) Relative HHLA2 (D) and c-Met (E) mRNA expression in five HCC organoids determined by qRT-PCR. (F) Representative images showing patient-derived organoid (PDO) death in response to c-Met inhibitor PHA665752 treatment. Dead cells are stained red with propidium iodide (PI), and live cells are stained green with Calcein-AM.

Figure S1

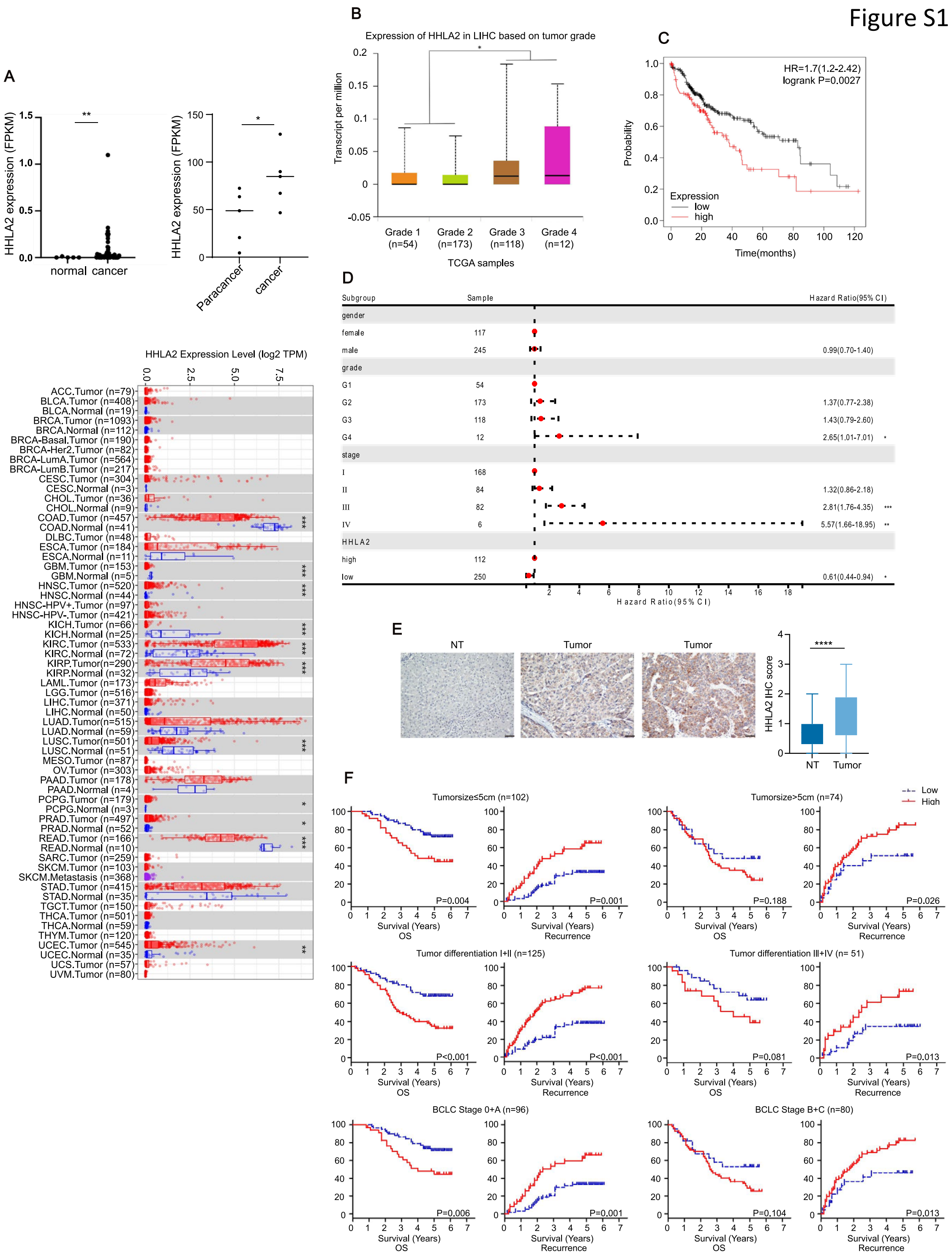

A
B
Expression of HHLA2 in LIHC based on tumor grade
C
HR=1.7(1.2-2.42)
logrank P=0.0027
D
E
F

Supplemental Figure 1. HHLA2 expression correlates with survival in patients with HCC. **(A)** Analysis of HHLA2 mRNA expression. *Top panels:* Comparison of HHLA2 mRNA levels in HCC versus Normal Liver tissues using data from Gene Expression Omnibus (GEO) datasets GSE64485 (n=50 HCC vs 5 Normal; left) and GSE190174 (n=5 HCC vs 5 Normal; right). P values were determined by Student's t-test. *Bottom panel:* Pan-cancer analysis displaying HHLA2 mRNA expression levels (log2 TPM) across various tumor types from The Cancer Genome Atlas (TCGA) and GTEx databases compared to corresponding available normal tissues (Data source: TIMER2.0). **(B)** HHLA2 mRNA expression (TPM) in HCC samples from the TCGA-LIHC project, stratified by pathological tumor grade (n = 371 individuals with grade information). P values determined by unpaired two-tailed t test **(C)** Kaplan-Meier analysis of overall survival (OS) in HCC patients from the TCGA-LIHC project (n = 371 individuals with survival data) stratified by high versus low HHLA2 mRNA expression (median cutoff). P value determined by log-rank test. **(D)** Forest plot showing multivariate Cox regression analysis of OS in the TCGA-LIHC cohort (analysis includes n = 357 individuals with complete data for all variables in the model). Hazard ratios (HR) and 95% confidence intervals (CI) are shown. P values determined by Wald's test. Patient data in (C-E) are from TCGA-LIHC project, n = 357 individuals with grade information. **(E)** HHLA2 protein levels assessed by immunohistochemical staining in HCC and paired normal tissues from the Guangdong cohort (n = 176 cases). P values were determined by paired two-tailed t test. **(F)** Kaplan-Meier survival curves for overall survival and recurrence-free Survival in the Guangdong HCC cohort (n = 176 individuals), stratified by tumor size (≤5 cm or >5 cm), tumor differentiation grade, and Barcelona Clinic Liver Cancer (BCLC) stage. P values were determined by log-rank test. * P < 0.05, ** P < 0.01, *** P < 0.001, **** P < 0.0001.

Figure S2

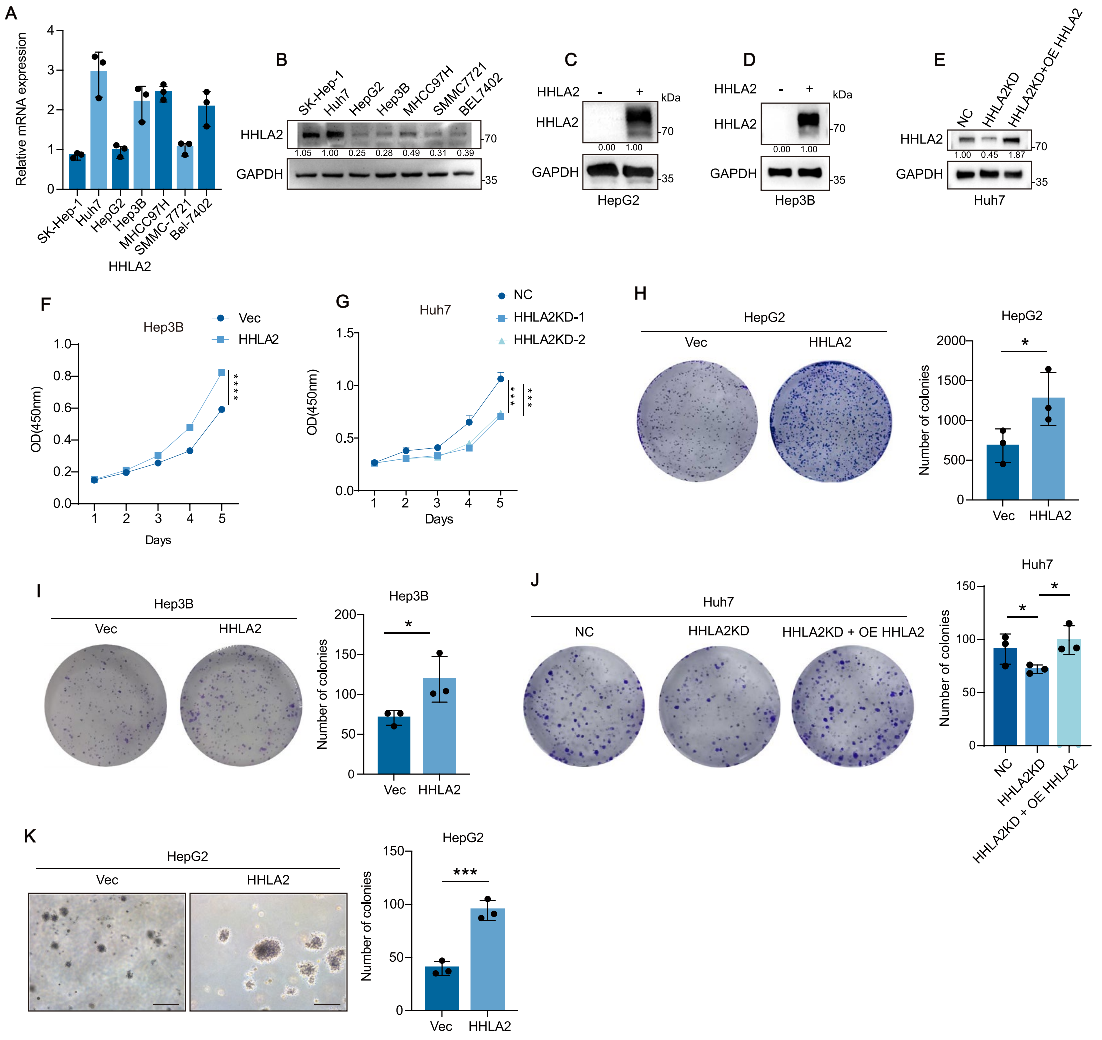

A
Relative mRNA expression
SK-Hep-1
Huh7
HepG2
Hep3B
MHCC97H
SMMC-7721
Bel-7402
HHLA2
B
SK-Hep-1
Huh7
HepG2
Hep3B
MHCC97H
SMMC7721
BEL7402
HHLA2
GAPDH
1.05 1.00 0.25 0.28 0.49 0.31 0.39
C
HHLA2 - +
kDa
HHLA2
GAPDH
0.00 1.00
HepG2
D
HHLA2 - +
kDa
HHLA2
GAPDH
0.00 1.00
Hep3B
E
NC
HHLA2KD
HHLA2KD+OE HHLA2
HHLA2
GAPDH
1.00 0.45 1.87
Huh7
F
Hep3B
Vec
HHLA2
OD(450nm)
Days
****
G
Huh7
NC
HHLA2KD-1
HHLA2KD-2
OD(450nm)
Days
***
***
H
HepG2
Vec
HHLA2
Number of colonies
*
I
Hep3B
Vec
HHLA2
Number of colonies
*
J
Huh7
NC
HHLA2KD
HHLA2KD + OE HHLA2
Number of colonies
*
*
K
HepG2
Vec
HHLA2
Number of colonies
***

Supplemental Figure 2. HHLA2 promotes HCC cell growth in vitro. (A, B) HHLA2 mRNA (A) and protein (B) expression in HCC cell lines. (C-E) Western blot validation of HHLA2 overexpression in HepG2 (C) and Hep3B (D)cells and HHLA2 knockdown in Huh7 (E) cells. All Western blots shown are representative of three independent experiments; relative quantification values are indicated below the bands, and full quantitative data/statistical analysis are provided in the supplementary Excel spreadsheet. (F, G) Cell proliferation assessed by CCK-8 assay in Hep3B (F)and Huh7 (G) cells with HHLA2 overexpression or knockdown. P values were determined by one-way ANOVA. (H-J) Two-dimensional colony formation assays in HepG2 (H), Hep3B (I), and Huh7 (J) cells with HHLA2 overexpression or knockdown (2 weeks). (K) Soft agar colony formation assay in HepG2-Vec and HepG2-HHLA2 cells. P values were determined by two-tailed Student's t test. * $P < 0.05$, ** $P < 0.01$, *** $P < 0.001$, **** $P < 0.0001$. Scale bars, 100 μm

Figure S3

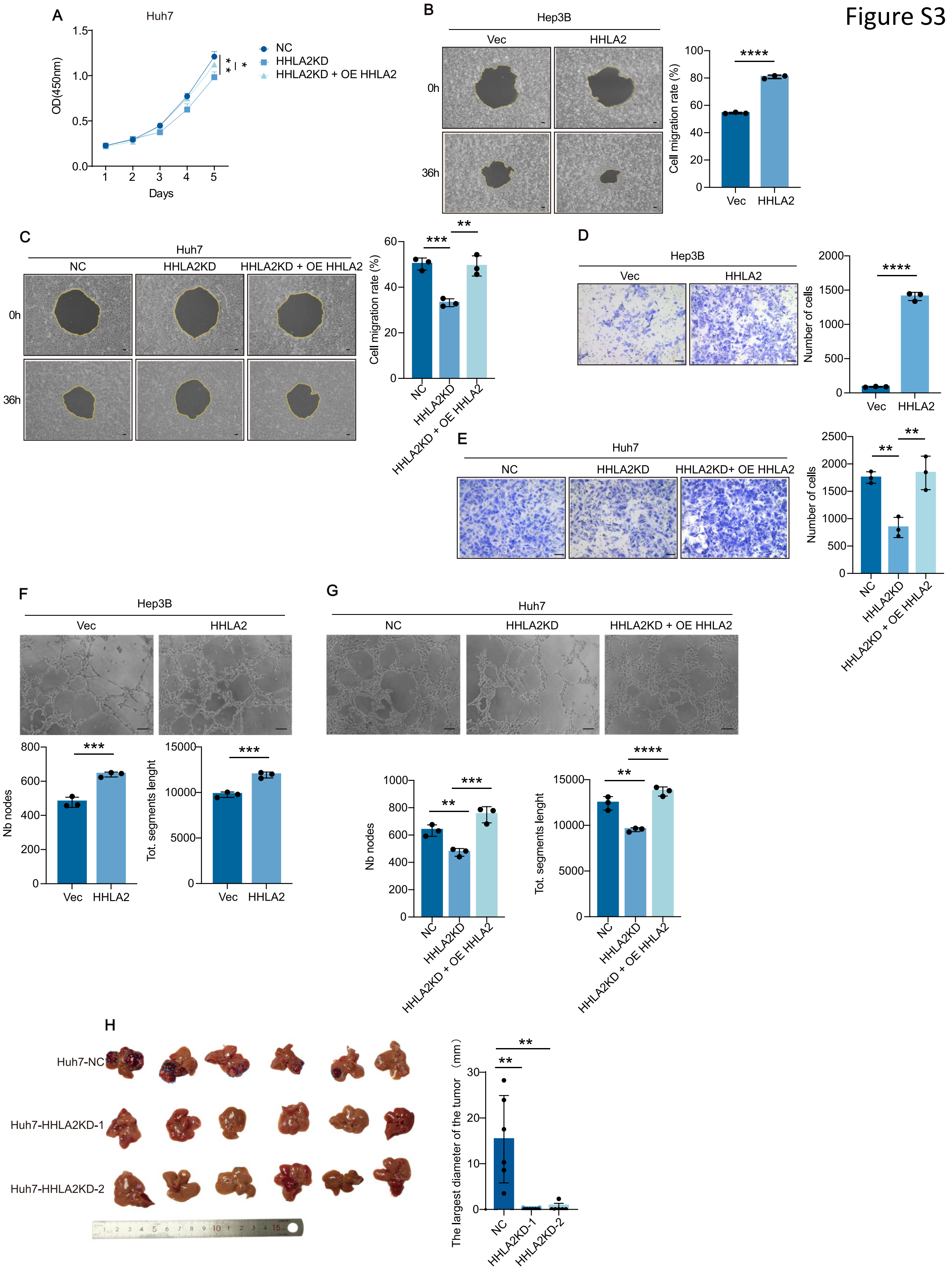
A
Huh7
OD(450nm)
Days
NC
HHLA2KD
HHLA2KD + OE HHLA2
B
Hep3B
Vec
HHLA2
0h
36h
Cell migration rate (%)
C
Huh7
NC
HHLA2KD
HHLA2KD + OE HHLA2
D
Hep3B
Number of cells
E
Huh7
HHLA2KD+ OE HHLA2
F
Nb nodes
Tot. segments lenght
G
H
Huh7-NC
Huh7-HHLA2KD-1
Huh7-HHLA2KD-2
The largest diameter of the tumor （mm）
HHLA2KD-1
HHLA2KD-2

Supplemental Figure 3. HHLA2 promotes aggressive phenotypes in HCC. (A) Cell proliferation assessed by CCK-8 assay in the indicated Huh7 cells. (B, C) Cell migration assays in Hep3B (B) and Huh7 (C) cells with HHLA2 overexpression or knockdown. (D, E) Transwell invasion assays in Hep3B (D) and Huh7 (E) cells with HHLA2 overexpression or knockdown. (F, G) HUVEC tube formation assays using conditioned media from Hep3B (F) or Huh7 (G) cells with HHLA2 overexpression or knockdown. (H) Representative images and quantification of liver tumor sizes in orthotopic xenografts derived from Huh7 cells expressing either a nontargeting shRNA (Huh7-shNC) or an shRNA targeting HHLA2 (Huh7-HHLA2KD). n = 6 mice per group. P values were determined by one-way ANOVA (A) or two-tailed Student's t test (B–H). * $P < 0.05$, ** $P < 0.01$, *** $P < 0.001$, **** $P < 0.0001$. Scale bars, 100 μm

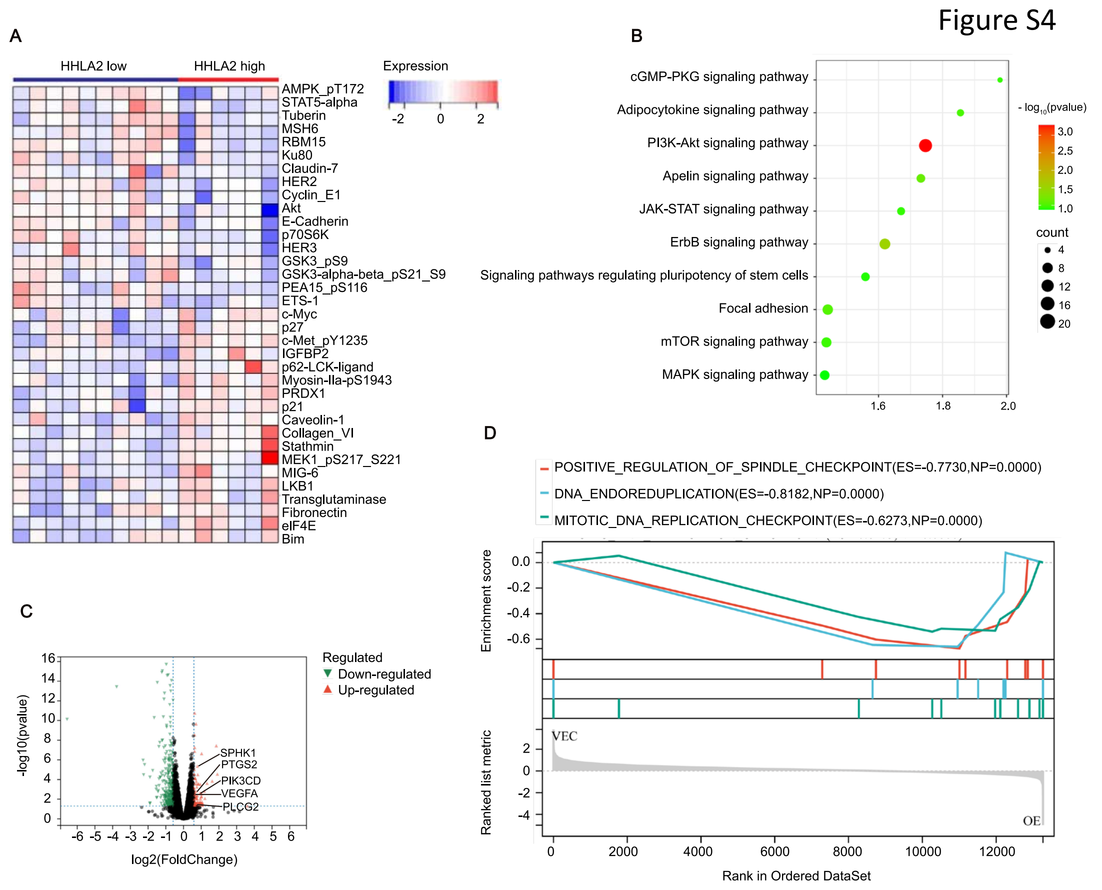

Supplemental Figure 4. HHLA2 expression modulates specific proteins and pathways. (A) Heatmap analysis of differentially expressed proteins and phosphoproteins in HCC samples with high or low HHLA2 expression from TCGA and The Cancer Proteome Atlas (TCPA) databases. The heatmap displays proteins and phosphoproteins with a fold change greater than 1.2 between high and low HHLA2–expressing HCC samples. (B) Kyoto Encyclopedia of Genes and Genomes (KEGG) pathway enrichment analysis of differentially expressed proteins identified in (A). Only pathways with $P < 0.05$ are shown. (C) Volcano plot of differentially expressed mRNAs in HepG2 cells following HHLA2 overexpression. Genes with |log2 (fold change) | > 0.4 and $P < 0.05$ are highlighted (n = 3 samples per group). (D) Gene set enrichment analysis (GSEA) of cell proliferation processes. GSEA was performed on the differentially expressed genes identified in (C). NES, normalized enrichment score.

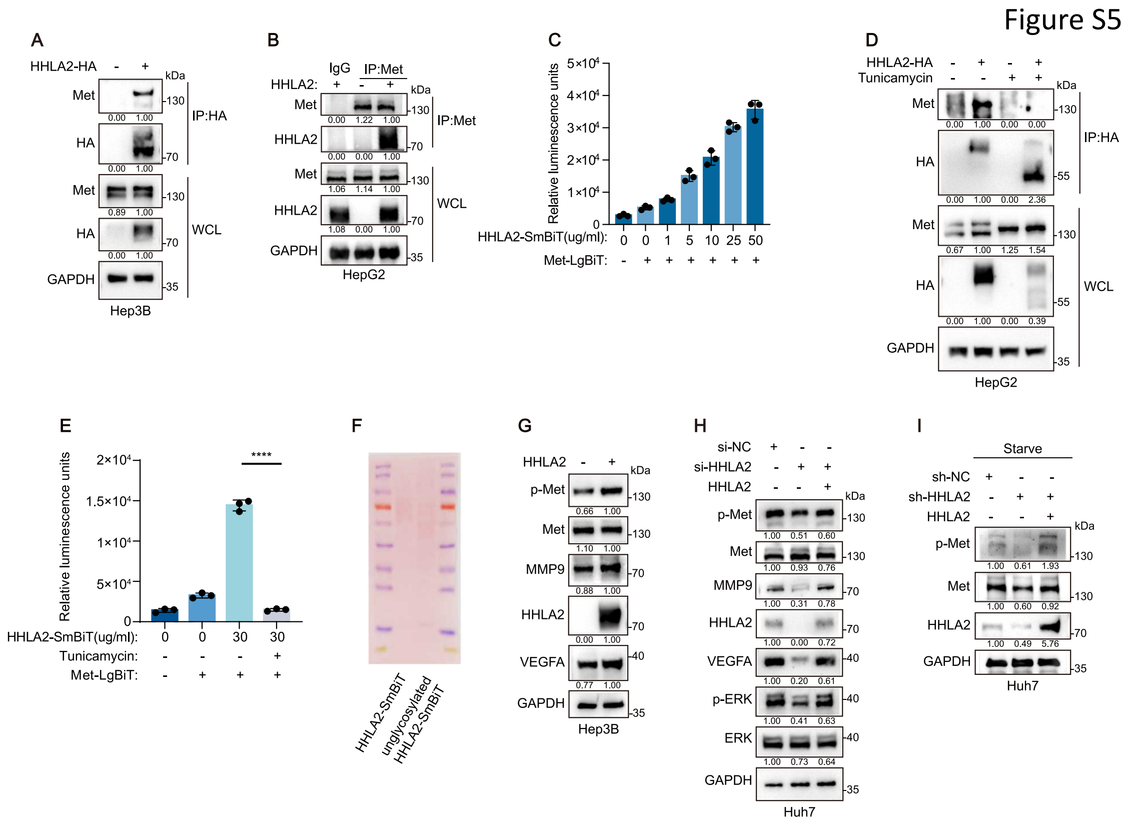
Figure S5
A
HHLA2-HA
Met
HA
IP:HA
WCL
GAPDH
Hep3B
B
IgG
IP:Met
HHLA2:
Met
HHLA2
IP:Met
WCL
GAPDH
HepG2
C
Relative luminescence units
HHLA2-SmBiT(ug/ml):
Met-LgBiT:
D
HHLA2-HA
Tunicamycin
Met
HA
IP:HA
WCL
GAPDH
HepG2
E
Relative luminescence units
****
HHLA2-SmBiT(ug/ml):
Tunicamycin:
Met-LgBiT:
F
HHLA2-SmBiT
unglycosylated HHLA2-SmBiT
G
HHLA2
p-Met
Met
MMP9
HHLA2
VEGFA
GAPDH
Hep3B
H
si-NC
si-HHLA2
HHLA2
p-Met
Met
MMP9
HHLA2
VEGFA
p-ERK
ERK
GAPDH
Huh7
I
Starve
sh-NC
sh-HHLA2
HHLA2
p-Met
Met
HHLA2
GAPDH
Huh7

Supplemental Figure 5. HHLA2 interacts directly with c-Met. (A) Coimmunoprecipitation of overexpressed HHLA2-HA and endogenous c-MET in Hep3B cells. (B) Coimmunoprecipitation of endogenous c-MET and HHLA2 in HepG2 cells. The antibodies used for immunoprecipitation (IP) and Western blot are indicated. (C) Split luciferase complementation assay to define the interaction domains between HHLA2 and c-Met. Luminescence was measured following the addition of indicated amounts of recombinant HHLA2-ExD SmBit-6His to 293-LgBiT-MET cells. (D) Coimmunoprecipitation of HHLA2-HA with endogenous c-Met in HepG2-Vec or HepG2-HHLA2 cells treated with 1 μg/ml tunicamycin (N-linked glycosylation inhibitor) or DMSO (control) for 24 hours. (E, F) In vitro interaction between N-linked glycosylated or unglycosylated HHLA2 and c MET assessed by split luciferase complementation assay. Luminescence was measured following the addition of indicated concentrations of glycosylated or unglycosylated HHLA2-ExD-SmBit-6His to 293-LgBiT-HHLA2 or 293-LgBiT-MET cells, respectively (E). P values were determined by two-tailed Student's t test. **** $P < 0.0001$. Ponceau S staining of input HHLA2-ExD-SmBit-6His proteins is shown in F. (G-I) Western blot analysis of c-MET signaling pathway proteins in Hep3B cells with or without HHLA2 overexpression (G) and Huh7 cells with or without HHLA2 knockdown and rescue under normal (H) or starvation (I) conditions. All Western blots shown are representative of three independent experiments; relative quantification values are indicated below the bands, and full quantitative data/statistical analysis are provided in the supplementary Excel spreadsheet.

Figure S6

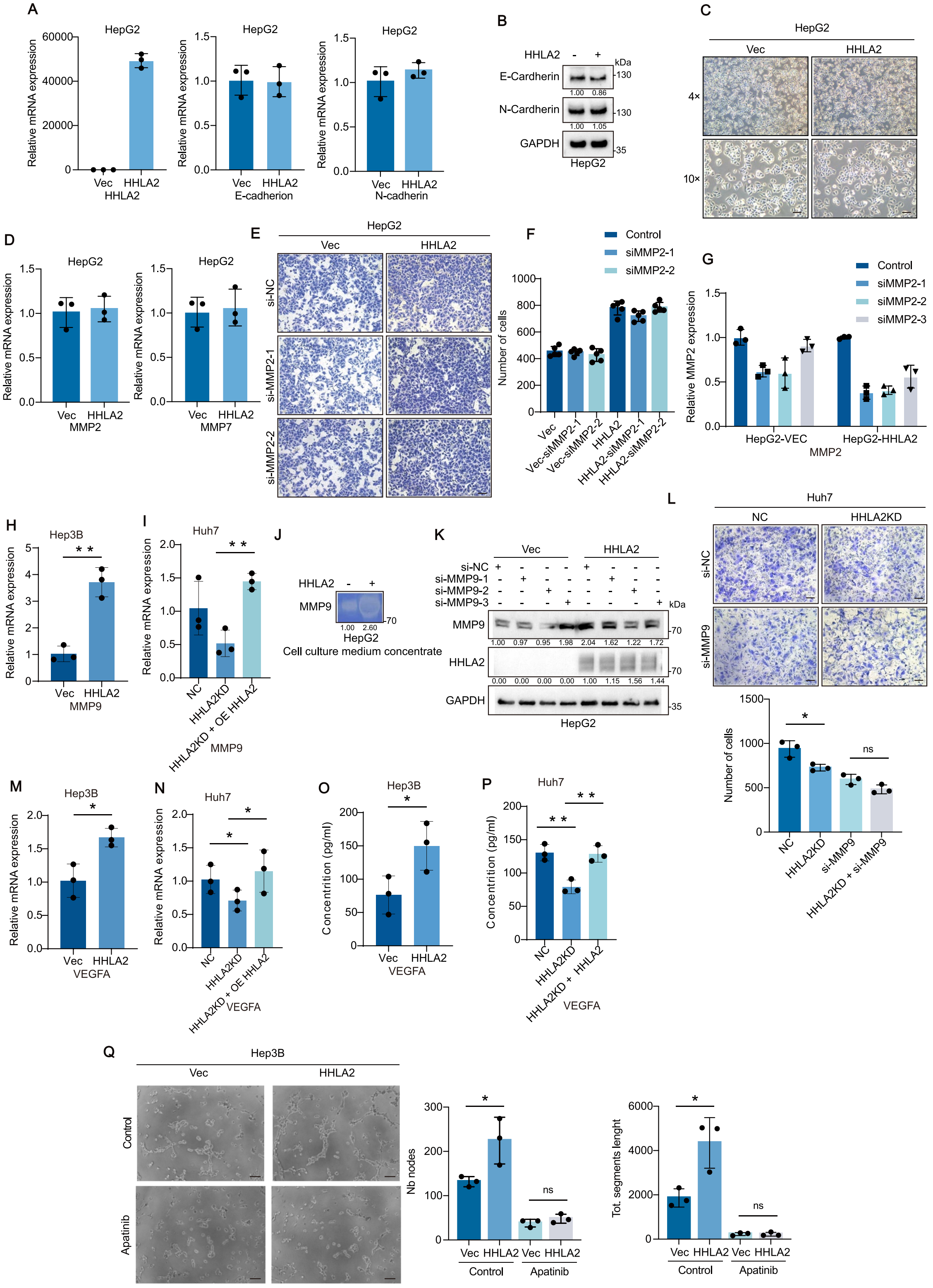
A
HepG2
Relative mRNA expression
Vec HHLA2
HHLA2
E-cadherion
N-cadherin
B
HHLA2
E-Cardherin
N-Cardherin
GAPDH
kDa
HepG2
C
Vec
4×
10×
D
MMP2
MMP7
E
si-NC
si-MMP2-1
si-MMP2-2
F
Control
siMMP2-1
siMMP2-2
Number of cells
G
siMMP2-3
Relative MMP2 expression
HepG2-VEC
HepG2-HHLA2
H
Hep3B
MMP9
I
Huh7
NC
HHLA2KD
HHLA2KD + OE HHLA2
J
MMP9
Cell culture medium concentrate
K
si-MMP9-1
si-MMP9-2
si-MMP9-3
L
si-MMP9
HHLA2KD + si-MMP9
M
VEGFA
N
O
Concentrition (pg/ml)
P
HHLA2KD + HHLA2
Q
Control
Apatinib
Nb nodes
Tot. segments lenght
ns

Supplemental Figure 6. MMP family and VEGFA-VEGFR2 signaling contribute to HHLA2-driven invasion and tumor angiogenesis. (A–D) qRT-PCR analysis of HHLA2, E-cadherin, and N-cadherin mRNA expression (A); Western blot analysis of the indicated protein levels (B); representative bright-field images showing morphology (C); and qRT-PCR analysis of MMP2 mRNA expression (D) in HepG2-Vec and HepG2-HHLA2 cells. (E–G) Representative images (E) and quantification (F) of invading HepG2-Vec and HepG2-HHLA2 cells with or without MMP2 knockdown; MMP2 knockdown efficacy was assessed by qRT-PCR (G). (H, I) qRT-PCR analysis of MMP9 mRNA expression in Hep3B-Vec and Hep3B-HHLA2 cells (H) and Huh7 cells with or without HHLA2 knockdown and rescue (I). **(J)** Gelatin zymography analysis of conditioned media from HepG2-Vec and HepG2-HHLA2 cells, demonstrating increased MMP9 enzymatic activity upon HHLA2 overexpression. **(K)** Western blot validation of MMP9 knockdown in HepG2-Vec and HepG2 HHLA2 cells. **(L)** Transwell invasion assays of Huh7 and Huh7-HHLA2KD cells with or without MMP9 knockdown. **(M, N)** qRT-PCR analysis of VEGFA mRNA expression in Hep3B cells with or without HHLA2 overexpression (M) and Huh7 cells with or without HHLA2 knockdown and rescue (N). **(O, P)** ELISA analysis of VEGFA secretion in Hep3B cells with or without HHLA2 overexpression (O) and Huh7 cells with or without HHLA2 knockdown and rescue (P). **(Q)** HUVEC tube formation assays using conditioned media from Hep3B Vec or Hep3B-HHLA2 cells, with or without treatment with 50 ng/ml apatinib (VEGF receptor inhibitor). Western blots and Gelatin zymography shown are representative of three independent experiments; relative quantification values are indicated below the bands, and full quantitative data/statistical analysis are provided in the supplementary Excel spreadsheet. P values were determined by two-tailed Student's t test. * $P < 0.05$, ** $P < 0.01$, *** $P < 0.001$, **** $P < 0.0001$. Scale bars, 100 μm.

Figure S7

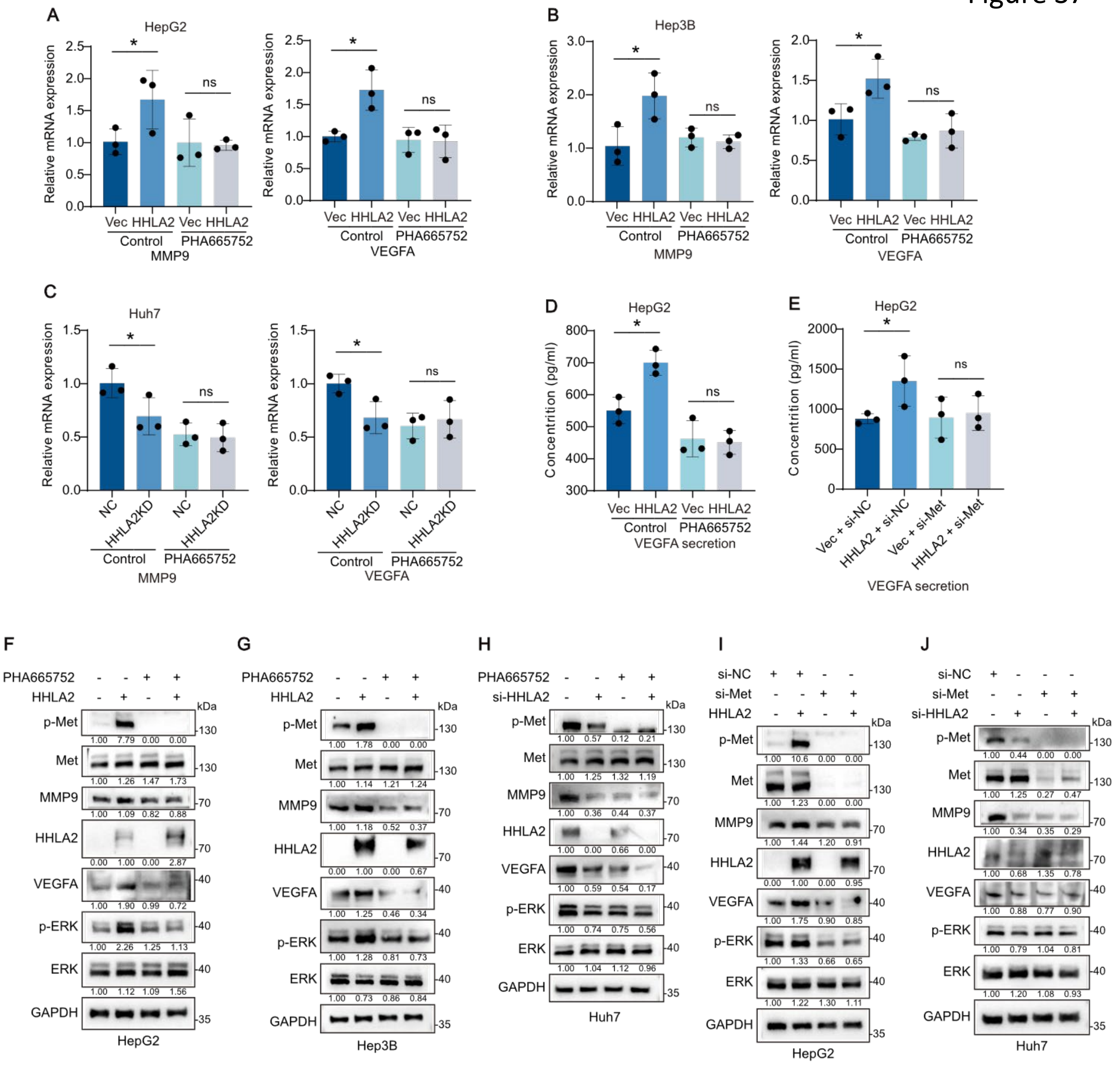
A
HepG2
Relative mRNA expression
Vec HHLA2 Vec HHLA2
Control PHA665752
MMP9
VEGFA
B
Hep3B
C
Huh7
NC HHLA2KD NC HHLA2KD
D
HepG2
Concentrition (pg/ml)
VEGFA secretion
E
Vec + si-NC
HHLA2 + si-NC
Vec + si-Met
HHLA2 + si-Met
F
G
H
I
J
PHA665752
HHLA2
si-HHLA2
si-NC
si-Met
p-Met
Met
MMP9
HHLA2
VEGFA
p-ERK
ERK
GAPDH
kDa
HepG2
Hep3B
Huh7

Supplemental Figure 7. c-Met activation is required for HHLA2-mediated MMP9 and VEGFA expression and secretion. (A–C) qRT-PCR analysis of MMP9 and VEGFA mRNA expression in (A) HepG2-Vec and HepG2-HHLA2 cells, (B) Hep3B-Vec and Hep3B-HHLA2 cells, and (C) Huh7-Vec and Huh7-HHLA2KD cells. All cell lines were treated with or without 100 nM PHA665752 (c-Met inhibitor). (D, E) ELISA of VEGFA secretion in HepG2-Vec and HepG2-HHLA2 cells with or without 100 nM PHA665752 treatment (D) and with or without c MET knockdown (E). (F–H) Western blot analysis of ERK activation, VEGFA, and MMP9 expression in (F) HepG2-Vec and HepG2-HHLA2 cells, (G) Hep3B-Vec and Hep3B-HHLA2 cells, and (H) Huh7-Vec and Huh7 HHLA2KD cells. All cell lines were treated with or without 100 nM PHA665752. (I, J) Western blot analysis of ERK activation, VEGFA, and MMP9 expression in (I) HepG2-Vec and HepG2-HHLA2 cells and (J) Huh7-Vec and Huh7-HHLA2KD cells. All cell lines were treated with or without c-MET knockdown. All Western blots shown are representative of three independent experiments; relative quantification values are indicated below the bands, and full quantitative data/statistical analysis are provided in the supplementary Excel spreadsheet. P values were determined by two-tailed Student's t test. ns, not significant; * $P < 0.05$, ** $P < 0.01$, *** $P < 0.001$, **** $P < 0.0001$. Scale bars, 100 μm

Figure S8

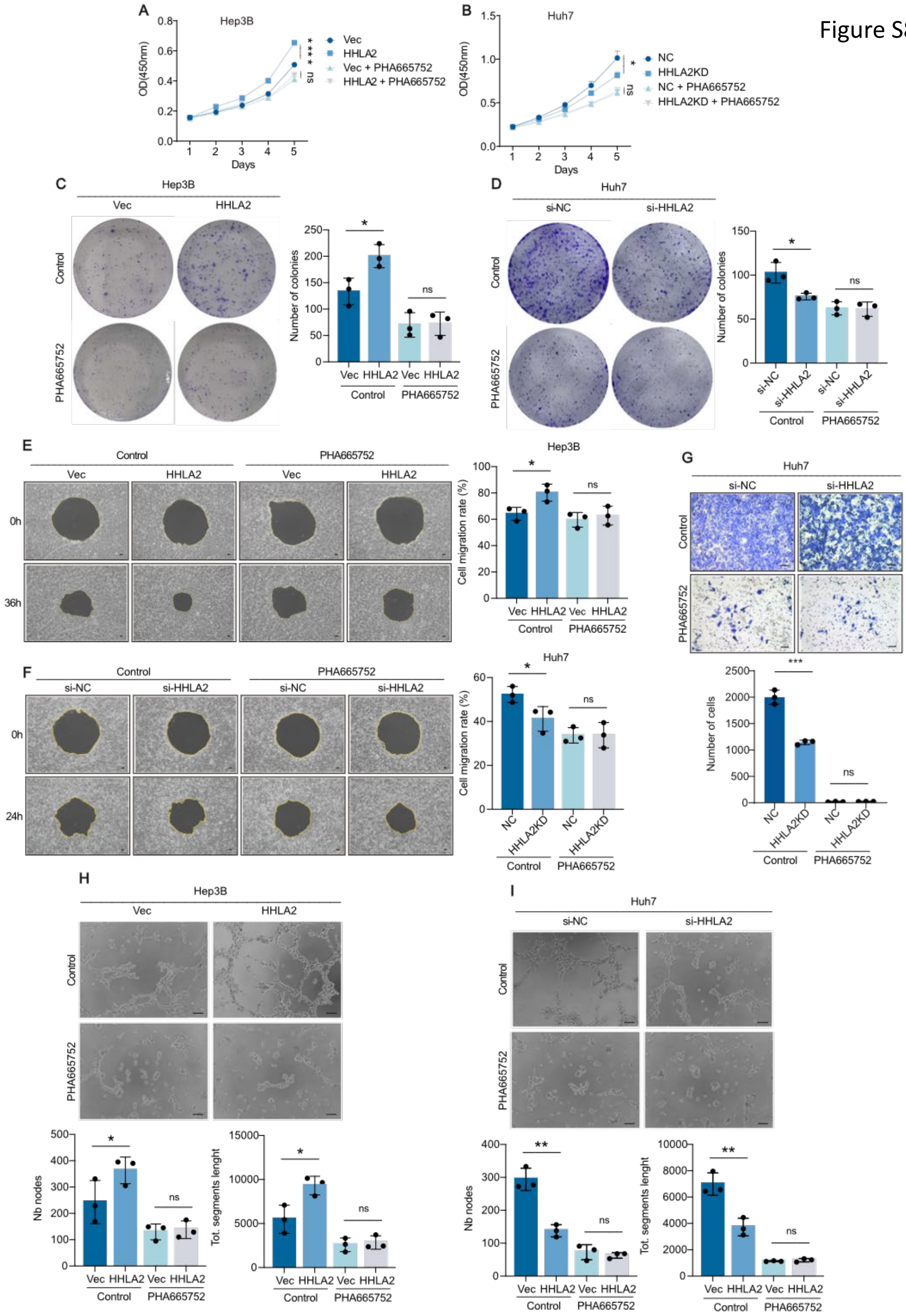
A
Hep3B
OD(450nm)
Days
Vec
HHLA2
Vec + PHA665752
HHLA2 + PHA665752
****
ns
B
Huh7
NC
HHLA2KD
NC + PHA665752
HHLA2KD + PHA665752
*
ns
C
Hep3B
Vec
HHLA2
Control
PHA665752
Number of colonies
D
Huh7
si-NC
si-HHLA2
E
Control
PHA665752
0h
36h
Hep3B
Cell migration rate (%)
F
24h
Huh7
NC
HHLA2KD
G
Number of cells
***
H
Nb nodes
Tot. segments lenght
I
**
ns

Supplemental Figure 8. c-Met inhibition by PHA665752 abrogates HHLA2-mediated HCC progression in vitro. (A, B) Cell proliferation assessed by CCK-8 assay in HepB3-Vec and HepB3-HHLA2 cells (A) or Huh7-Vec and Huh7-HHLA2KD cells (B). (C, D) Two-dimensional colony formation assay in HepB3-Vec and HepB3-HHLA2 cells (C) or Huh7-Vec and Huh7-HHLA2KD cells (D). (E, F) Cell migration assay in HepB3-Vec and HepB3 HHLA2 cells (E) or Huh7-Vec and Huh7-HHLA2KD cells (F). (G) Transwell invasion assay of Huh7-Vec and Huh7-HHLA2KD cells. (H, I) HUVEC tube formation assay induced by conditioned media from HepB3-Vec and HepB3-HHLA2 cells (H) or Huh7-Vec and Huh7-HHLA2KD cells (I). All cells were treated with 100 nM PHA665752 or DMSO as indicated. P values were determined by two-tailed Student's t test. ns, not significant; * $P < 0.05$, ** $P < 0.01$, *** $P < 0.001$, **** $P < 0.0001$. Scale bars, 100 μm.

Figure S9

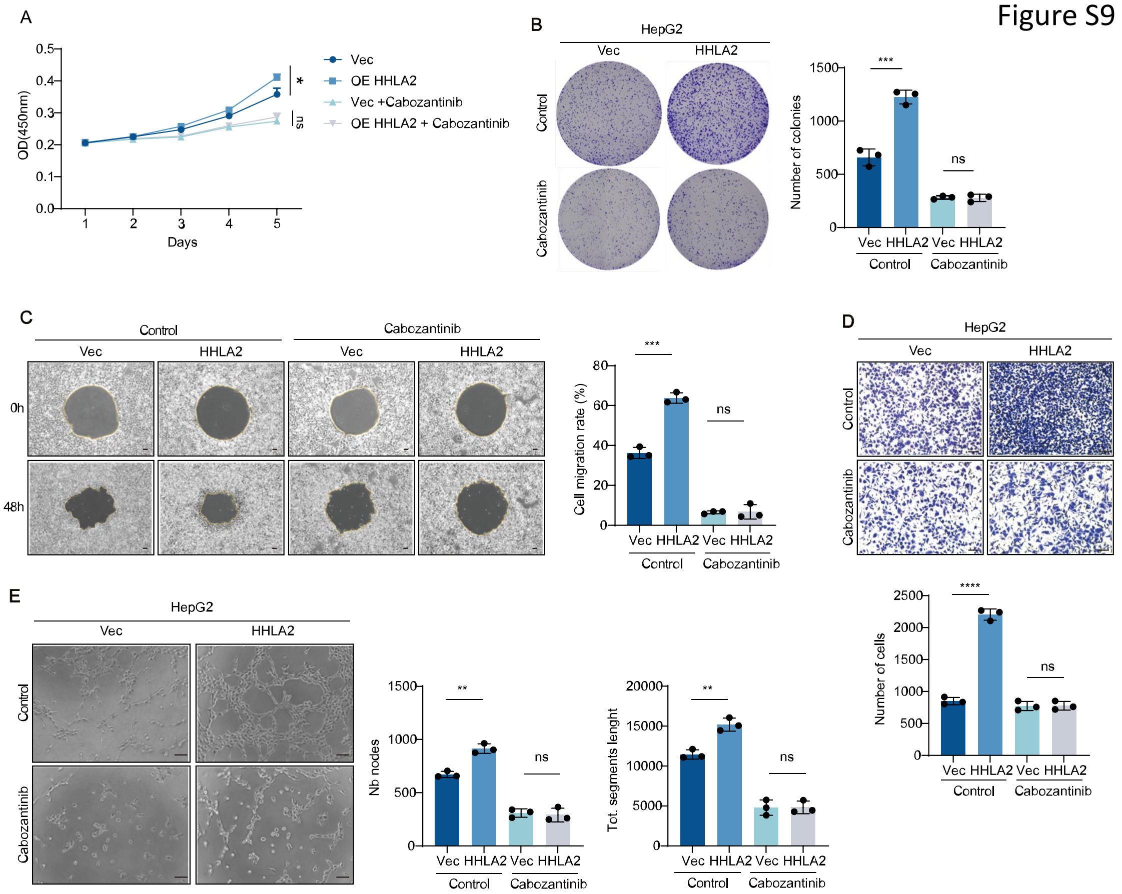

Supplemental Figure 9. Clinically relevant c-Met inhibitor cabozantinib abrogates HHLA2-mediated aggressive phenotypes *in vitro*. (A-E) Assessment of malignant phenotypes in HepG2-Vec and HepG2-HHLA2 cells, with or without 1 μM cabozantinib treatment. Phenotypes evaluated include (A) cell proliferation by CCK-8 assay, (B) two-dimensional colony formation, (C) cell migration assay using a wound-healing approach, (D) Transwell invasion assay, and (F) HUVEC tube formation using conditioned media from treated HepG2 cells. P values were determined by one-way ANOVA for (A), or two-tailed t test for (B-E). * $P < 0.05$, ** $P < 0.01$, *** $P < 0.001$, **** $P < 0.0001$. Scale bars, 100 μm.

Figure S10

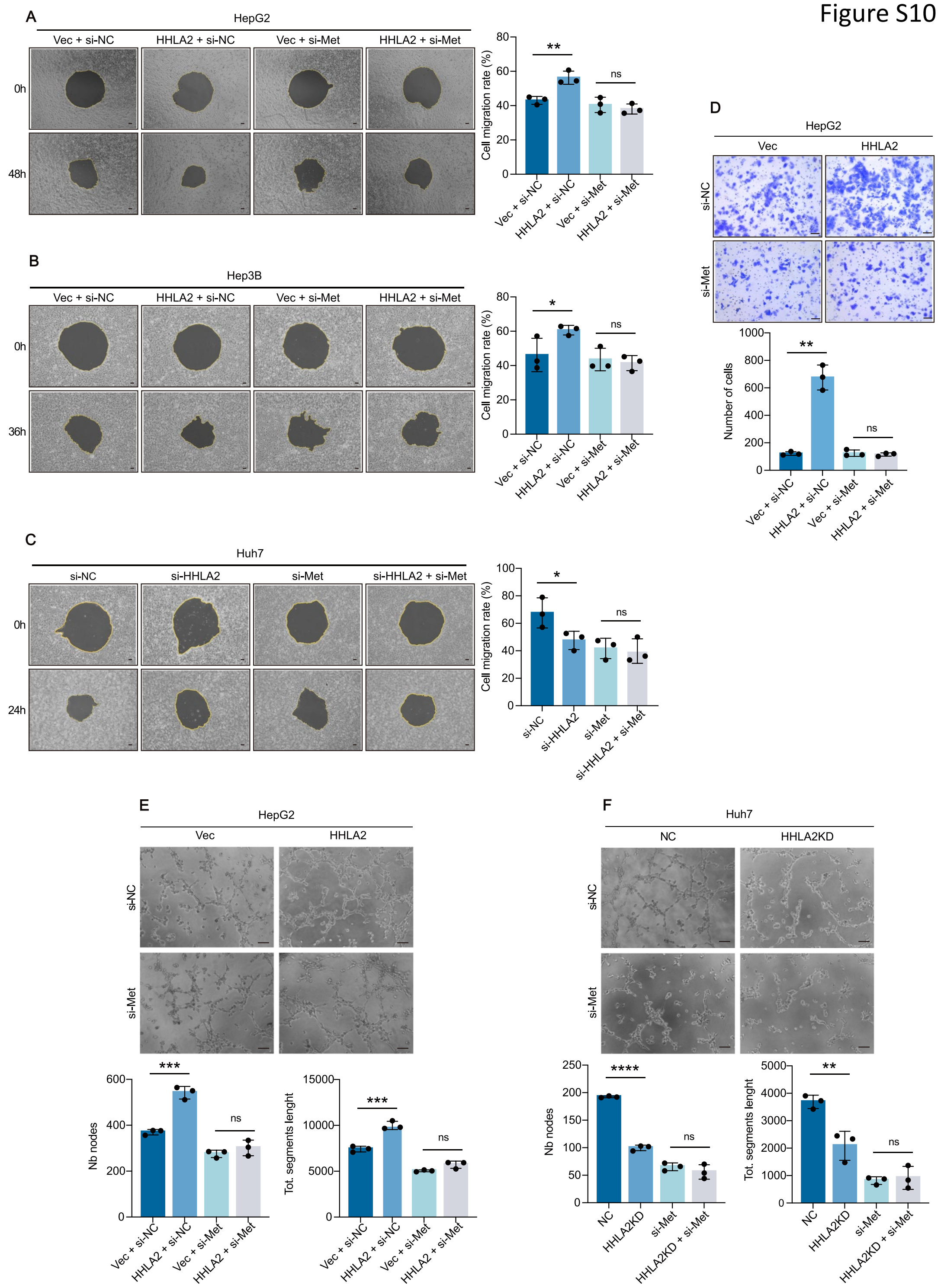

A
HepG2
Vec + si-NC
HHLA2 + si-NC
Vec + si-Met
HHLA2 + si-Met
0h
48h
Cell migration rate (%)
**
ns
B
Hep3B
Vec + si-NC
HHLA2 + si-NC
Vec + si-Met
HHLA2 + si-Met
0h
36h
Cell migration rate (%)
*
ns
C
Huh7
si-NC
si-HHLA2
si-Met
si-HHLA2 + si-Met
0h
24h
Cell migration rate (%)
*
ns
D
HepG2
Vec
HHLA2
si-NC
si-Met
Number of cells
**
ns
E
HepG2
Vec
HHLA2
si-NC
si-Met
Nb nodes
Tot. segments lenght
***
ns
F
Huh7
NC
HHLA2KD
si-NC
si-Met
Nb nodes
Tot. segments lenght
****
**
ns
NC
HHLA2KD
si-Met
HHLA2KD + si-Met

Supplemental Figure 10. c-Met knockdown abrogates HHLA2-mediated HCC progression in vitro. (A C) Cell migration assays of HepG2-Vec and HepG2-HHLA2 cells (A), HepB3-Vec and HepB3-HHLA2 cells (B), or Huh7-Vec and Huh7-HHLA2KD cells (C) with or without c-MET knockdown. (D) Transwell invasion assay of HepG2-Vec and HepG2-HHLA2 cells with or without c-MET knockdown. (E, F) HUVEC tube formation assays induced by conditioned media from HepG2-Vec and HepG2-HHLA2 cells (E) or Huh7-Vec and Huh7-HHLA2KD cells (F) with or without c-MET knockdown. P values were determined by two-tailed Student's t test. ns, not significant; * $P < 0.05$, ** $P < 0.01$, **** $P < 0.0001$. Scale bars, 100 μm

Supplemental Figure 11. Infiltration of immune cell subsets in mouse tumor tissue. (A) Representative images of orthotopic liver tumors in C57BL/6 mice following HDTVi delivery of myc-HHLA2, a control vector, wild type (WT) c-MET, or kinase-dead (KD) c-MET. (A) Representative images of liver tumors in orthotopic xenograft models in nude mice injected with HepG2-HHLA2 cells, followed by intraperitoneal administration of PHA665752 (n = 5 mice per group). (B) Representative images of lung tissues from nude mice following tail vein injection of HepG2-Vec or HepG2-HHLA2 cells, with or without PHA665752 treatment every other day for 28 days. Lung tissues were assessed for HCC metastasis. (C-E) Flow cytometry analysis of immune cell subpopulations in C57BL/6 mouse livers following HDTVi delivery of Myc-HHLA2 or a control vector, along with N-RasV12/myr AKT1 and the Sleeping Beauty transposon system. (C) Representative flow cytometry plots showcasing various immune cell populations (macrophages, neutrophils, CD4+ T cells, CD8+ T cells, and B cells) in the liver. (D) Quantification of the relative abundance of each immune cell subpopulation. (E) Changes in indicated immune cell activity in different treatment groups. Data are presented as mean ± SD (n = 5 mice per group). P values were determined by two-tailed Student's t test. ns, not significant; * $P < 0.05$, ** $P < 0.01$, **** $P < 0.0001$. Scale bars, 100 μm

Figure S11

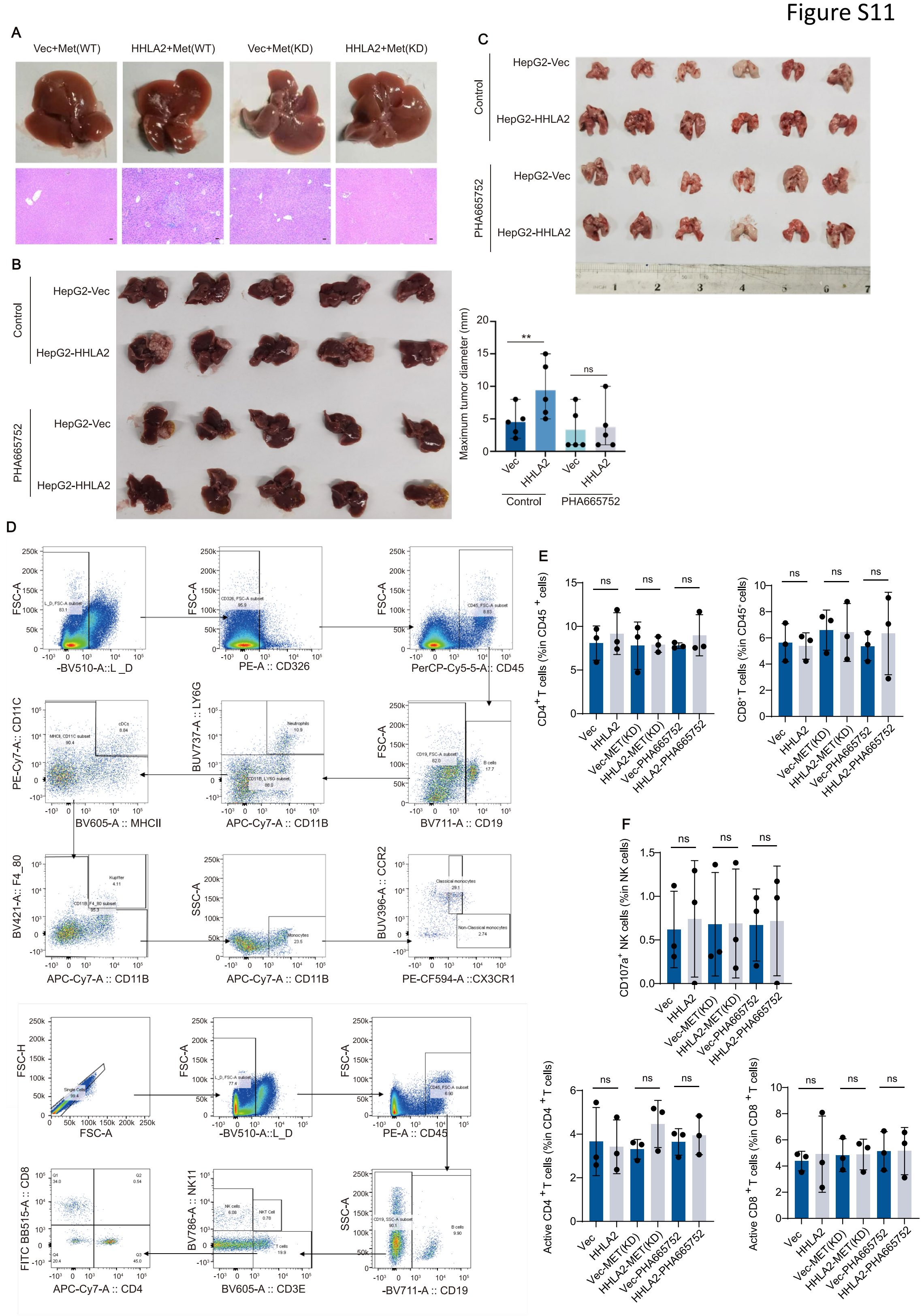
A
Vec+Met(WT)
HHLA2+Met(WT)
Vec+Met(KD)
HHLA2+Met(KD)
B
Control
PHA665752
HepG2-Vec
HepG2-HHLA2
Maximum tumor diameter (mm)
**
ns
Vec
HHLA2
C
D
-BV510-A::L_D
PE-A :: CD326
PerCP-Cy5-5-A:: CD45
PE-Cy7-A:: CD11C
BV605-A :: MHCII
BUV737-A :: LY6G
APC-Cy7-A :: CD11B
BV711-A :: CD19
BV421-A:: F4_80
SSC-A
BUV396-A :: CCR2
PE-CF594-A ::CX3CR1
FSC-H
FSC-A
PE-A :: CD45
FITC BB515-A :: CD8
APC-Cy7-A :: CD4
BV786-A :: NK11
BV605-A :: CD3E
-BV711-A :: CD19
E
CD4+ T cells (%in CD45 + cells)
CD8+ T cells (%in CD45+ cells)
F
CD107a+ NK cells (%in NK cells)
Active CD4 + T cells (%in CD4 + T cells)
Active CD8 + T cells (%in CD8 + T cells)
Vec
HHLA2
Vec-MET(KD)
HHLA2-MET(KD)
Vec-PHA665752
HHLA2-PHA665752

Figure S12

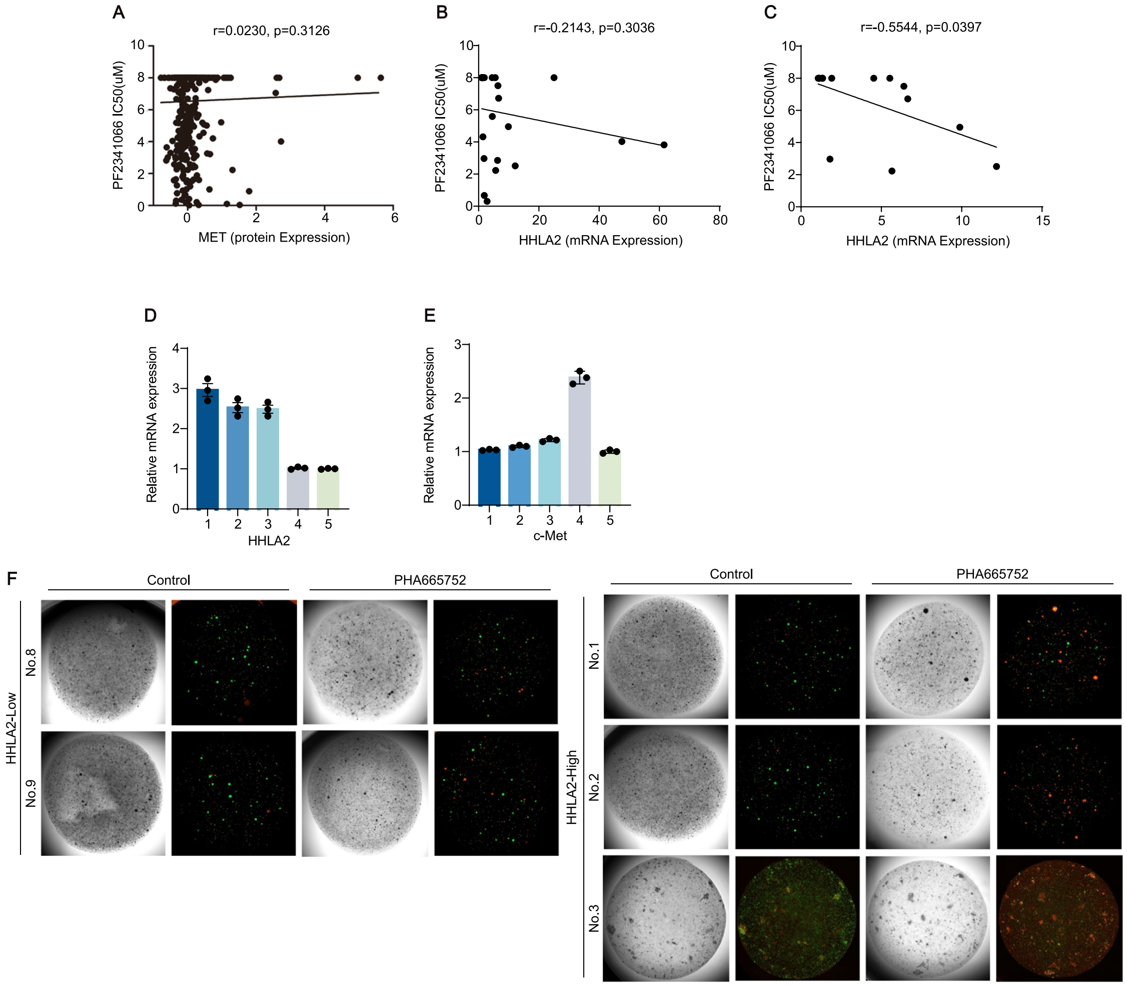

Supplemental Figure 12. HHLA2 expression predicts efficacy of c-Met inhibitor therapy in HCC. (A–C) Correlation between c-Met/HHLA2 expression and c-Met inhibitor efficacy in HCC cell lines. Relationship between c-Met expression level (A), HHLA2 expression level (B), or HHLA2 expression levels with abnormal c Met expression. Scoring methods are detailed in the Supplemental Methods section. P value was determined by Pearson correlation analysis. P < 0.0001. (C) and the efficacy of the c-Met inhibitor PF2341066 in cancer cell lines from the Cancer Cell Line Encyclopedia (CCLE) database. (D, E) Relative HHLA2 (D) and c-Met (E) mRNA expression in five HCC organoids determined by qRT-PCR. (F) Representative images showing patient-derived organoid (PDO) death in response to c-Met inhibitor PHA665752 treatment. Dead cells are stained red with propidium iodide (PI), and live cells are stained green with Calcein-AM.
